# Integrative Multi-Omics and Pan-Cancer Analyses Identify CCL20 as a Prognostic Biomarker with Therapeutic Relevance in Esophageal Cancer

**DOI:** 10.3390/biomedicines14051062

**Published:** 2026-05-07

**Authors:** Shixiang Guo, Xiaoyu Chen, Mingfeng Wei, Fuzhi Yang, Shuai Jiang, Liting Zhao, Lefei Hu, Zheng Li, Xiaoyong Shen

**Affiliations:** 1 Shanghai Key Laboratory of Clinical Geriatric Medicine, Affiliated Huadong Hospital, Fudan University, Shanghai 200040, China; 24211280027@m.fudan.edu.cn (S.G.); yangfuzhi0314@126.com (F.Y.); lefeihu315@163.com (L.H.); 2 Department of Thoracic Surgery, Affiliated Huadong Hospital, Fudan University, Shanghai 200040, China; billcxy1217@163.com (X.C.); 13027635105@163.com (M.W.); jsdanxtandkao@163.com (S.J.); 3 Department of General Surgery, Affiliated Huadong Hospital, Fudan University, Shanghai 200040, China; tingting0726@126.com

**Keywords:** CCL20, esophageal cancer, pan-cancer analysis, immune microenvironment, single-cell RNA sequencing, therapeutic target

## Abstract

**Background**: CCL20 is a key chemokine involved in tumor-associated inflammation and immune microenvironment remodeling, but its biological and clinical relevance in esophageal cancer (ESCA) and across cancers remains incompletely defined. This study aimed to systematically characterize the expression pattern, prognostic value, immune associations, and potential translational relevance of CCL20 using an integrative multi-omics framework. **Methods**: The biological and clinical significance of CCL20 was investigated through differential expression analysis, weighted gene co-expression network analysis, survival and clinicopathological association analyses, ROC-based diagnostic evaluation, immune infiltration and tumor microenvironment characterization, immune checkpoint correlation analysis, single-cell transcriptomic analysis, in silico knockout analysis, drug sensitivity prediction, molecular docking, and tissue microarray-based immunohistochemistry. **Results**: CCL20 was aberrantly upregulated in ESCA and multiple other solid tumors, and its high expression was associated with poor prognosis in several cancer types. Tissue microarray-based immunohistochemistry further confirmed CCL20 overexpression at the protein level in ESCA. In the TCGA cohort, CCL20 showed favorable diagnostic performance and was associated with poorer survival outcomes in ESCA. High CCL20 expression was also closely associated with immunoregulatory infiltration patterns, increased immune checkpoint expression, and enhanced stromal features. Single-cell analysis showed that CCL20 was predominantly expressed in monocytes/macrophages and may mediate immune communication between myeloid and lymphoid/dendritic cells through the CCL20–CCR6 axis. In silico knockout analysis further suggested that CCL20 depletion perturbs inflammation-, chemotaxis-, and immune regulation-related transcriptional programs in monocytes/macrophages. Drug sensitivity prediction and molecular docking provided preliminary clues supporting the therapeutic relevance of the CCL20 axis. **Conclusions**: CCL20 may represent a biologically relevant candidate biomarker in ESCA, with potential diagnostic, prognostic, and therapeutic relevance.

## 1. Introduction

Esophageal cancer (ESCA) is one of the most burdensome malignancies of the digestive tract worldwide, characterized by high incidence and mortality rates and an overall poor prognosis. Over the past two decades, substantial advances have been made in cancer treatment, including surgery, chemoradiotherapy, targeted therapy, and immunotherapy; however, overall prognostic improvement in esophageal cancer has remained limited, and clinical management is still challenging because advanced disease is associated with high rates of recurrence and metastasis and marked heterogeneity in treatment response [1,2,3,4]. Esophageal cancer exhibits marked molecular and histological heterogeneity, with distinct subtypes differing in driver events, signaling pathways, and immune ecosystems, which limits the utility of conventional single clinical indicators for precise stratification and treatment decision-making [2,3,4]. The tumor microenvironment (TME) is regarded as a key determinant of tumor progression, immune evasion, and immunotherapy response; in esophageal cancer in particular, immunosuppressive cell infiltration, dysregulated cytokine networks, and aberrant immune checkpoint expression jointly shape a complex therapeutic response landscape [3,5,6]. Therefore, identifying novel molecular biomarkers with both tumor-biological relevance and immune microenvironment-informative value is important for improving risk assessment and individualized intervention in esophageal cancer.

C-C motif chemokine ligand 20 (CCL20) is a pro-inflammatory chemokine increasingly implicated in tumor-promoting immune remodeling. Recent studies have shown that CCL20 is aberrantly upregulated in multiple solid tumors and contributes to immunosuppressive remodeling, stemness maintenance, and therapeutic resistance [7,8,9,10,11,12,13,14,15]. In metastatic prostate cancer, myeloid-derived CCL20 was identified as a key component of the immunosuppressive bone metastatic niche, and blockade of the CCL20-CCR6 axis restored T-cell activity and prolonged survival in preclinical models [7]. In breast cancer, tumor-derived CCL20 promoted PMN-MDSC-associated stem-like traits, and CCL20-related immune remodeling has also been linked to impaired antitumor immune surveillance and reduced anti-PD-1 efficacy [8,9]. In hepatocellular carcinoma, macrophage-derived CCL20 facilitated CCR6+ Treg recruitment and supported an immunosuppressive microenvironment [10]. Consistently, mechanistic studies further showed that the CCR6-CCL20 axis enhances regulatory T-cell glycolysis and immunosuppressive fitness in tumors [11]. Emerging evidence also supports a role for CCL20 in esophageal cancer. Recent high-dimensional single-cell proteomic analyses suggested that CCR4/CCR6-related chemokine features were associated with survival and neoadjuvant treatment response in esophageal squamous cell carcinoma [16], while independent studies linked CCL20 upregulation to Treg recruitment, immune dysregulation, and tumor progression [17,18]. However, the role of CCL20 in esophageal cancer remains incompletely defined, and current evidence is insufficient to provide an integrated understanding of its expression pattern, prognostic relevance, immune context, and potential translational value. To address this gap, we performed an integrative multi-omics analysis focused on CCL20 to systematically evaluate its expression characteristics, prognostic value, and immune associations in ESCA and across cancers, thereby providing a basis for future studies of CCL20 as a potential biomarker and candidate therapeutic target in esophageal cancer.

## 2. Materials and Methods

### 2.1. Differential Expression Analysis

Publicly available esophageal cancer (ESCA) transcriptomic microarray datasets GSE23400 (53 paired tumor and adjacent normal samples) and GSE38129 (30 paired tumor and adjacent normal samples) were obtained from the GEO database (https://www.ncbi.nlm.nih.gov/geo/, accessed on 1 February 2026). Probe IDs were mapped to gene symbols using the corresponding platform annotation files; probes without valid gene symbols or mapped to multiple genes were removed, and duplicate probes for the same gene were collapsed by averaging. The two gene-level matrices were merged on shared gene symbols, and dataset-origin batch effects were removed using removeBatchEffect in the limma package (version 3.62.2). Batch-correction performance was evaluated using boxplots and principal component analysis (PCA). A log2(x + 1) transformation was applied when indicated by the expression quantile distribution, followed by cross-sample normalization using the limma package. Differential expression analysis in the merged GEO discovery cohort (83 tumors and 83 normal tissues) was performed using limma, and genes with |log2FC| > 1 and Benjamini–Hochberg adjusted *p* values (false discovery rate, FDR) < 0.05 were defined as differentially expressed genes (DEGs).

### 2.2. Weighted Gene Co-Expression Network Analysis (WGCNA)

WGCNA was used to identify co-expression gene modules associated with esophageal cancer biology. A co-expression network was constructed using the candidate gene set obtained from differential expression analysis (DEGs). Hierarchical clustering was first performed to detect and exclude outlier samples. A soft-thresholding power of 14 was selected to satisfy the scale-free topology criterion (signed R^2^ ≥ 0.85). Based on the selected soft threshold, the adjacency matrix and topological overlap matrix (TOM) were calculated, and modules were identified using the dynamic tree cut method (minimum module size = 50 genes), followed by module merging according to module eigengene similarity (cutHeight = 0.25). Module eigengenes (MEs) were then calculated, and Pearson correlation analysis was used to assess the associations between modules and clinical phenotypes (tumor vs. normal), allowing the identification of key modules significantly related to esophageal cancer for downstream analyses.

### 2.3. Validation of TCGA Data

Genes from the key WGCNA module were intersected with the DEG set. To validate the expression patterns and prognostic relevance of intersecting genes, ESCA RNA-seq data were downloaded from TCGA via UCSC Xena (https://xena.ucsc.edu/, accessed on 1 February 2026), and matched clinical data were obtained from TCGA (https://www.cancer.gov/ccg/research/genome-sequencing/tcga, accessed on 1 February 2026). After quality control, samples with missing or abnormal data were removed, and expression values were normalized as log2(TPM + 1). Only intersecting genes present in the TCGA expression matrix were retained. Tumor–normal expression differences were assessed using the Wilcoxon rank-sum test, with Benjamini–Hochberg correction for multiple testing. Genes with |log2FC| > 1 and FDR < 0.05 were considered differentially expressed. In addition, the integrated TCGA–GTEx dataset was used to compare CCL20 transcript levels across cancer types.

### 2.4. External Validation and Biomarker Comparison

To further assess robustness, an independent external GEO cohort, GSE161533 (28 tumors and 28 normal tissues), was used for validation. Probe annotation, gene-level summarization, and limma-based differential analysis were performed using the same workflow and thresholds described for the discovery cohort. Diagnostic performance was evaluated by receiver operating characteristic (ROC) analysis using the pROC package. In addition, CCL20 was compared head-to-head with reported ESCA-related biomarkers (CEACAM5, KRT19, SERPINB3, SERPINB4, TP63, KRT7, MUC5AC, MUC6, CDX2, and AGR2) in the TCGA-ESCA cohort using a unified analytical framework that included unpaired tumor–normal comparison, paired comparison in matched samples, diagnostic ROC analysis, Kaplan–Meier survival analysis, univariable Cox regression, and 1-year time-dependent ROC analysis.

### 2.5. Prognosis Analysis

The prognostic value of candidate differentially expressed genes was evaluated in the TCGA cohort by dividing patients into high- and low-expression groups according to the median expression level; Kaplan–Meier survival analysis was performed using the survival package (version 3.7-0), intergroup survival differences were assessed by the log-rank test, and hazard ratios (HRs) with 95% confidence intervals were estimated using univariable Cox proportional hazards models. Only CCL20 met the predefined significance criteria in both the log-rank test and univariable Cox regression (*p* < 0.01) and was therefore defined as a prognosis-related gene. Receiver operating characteristic (ROC) curve analysis was further performed to evaluate the discriminatory performance of CCL20 expression for esophageal cancer.

### 2.6. Clinical Correlation Analysis

Sample IDs were standardized between the TCGA expression matrix and the clinical dataset. In tumor samples, CCL20 expression was compared across clinicopathological subgroups, including age, sex, tumor (T) stage, lymph node (N) stage, and overall pathological stage. A survival dataset was then constructed using follow-up time and outcome status. CCL20 expression and clinicopathological variables were included in Cox proportional hazards models, and univariable and multivariable analyses were performed using the survival package to evaluate independent prognostic associations.

### 2.7. Immune Infiltration Analysis

To investigate the relationship between immune infiltration patterns and CCL20 expression, we extracted the RNA-seq gene expression matrix from the TCGA database and performed quantile normalization using the preprocessCore package (version 1.68.0). The CIBERSORT algorithm was then applied to estimate the relative infiltration proportions of multiple immune cell subsets in each sample, and only samples with permutation *p* < 0.05 were retained for downstream analyses. We further implemented the ESTIMATE algorithm using the estimate package (version 1.0.13) to calculate StromalScore, ImmuneScore, and ESTIMATEScore for each patient and assessed the associations of CCL20 expression with immune cell infiltration levels and tumor microenvironment scores.

### 2.8. Anticancer Drug Sensitivity Analysis and Immunotherapy Analysis of CCL20 in ESCA

Based on the TCGA transcriptomic expression matrix and publicly available GDSC2 drug-response data (https://osf.io/c6tfx/files/osfstorage, accessed on 1 February 2026), the calcPhenotype function in the oncoPredict R package (version 1.2) was used, with GDSC cell lines as the training set, to estimate the predicted IC50 values of multiple anticancer drugs for each patient. Patients were then stratified into high- and low-CCL20 expression groups, and differences in drug sensitivity between groups were compared using the Wilcoxon rank-sum test; Pearson correlation coefficients between CCL20 expression and predicted IC50 values for each drug were further calculated. All drug sensitivity differences and correlation results were visualized using R packages including ggplot2, ggpubr, and pheatmap.

### 2.9. Molecular Docking Analysis of Candidate Anticancer Drugs with CCL20

Molecular docking and visualization were performed using AutoDock Vina (version 1.2.3) and Pymol (version 3.1.0) to evaluate the binding affinity between anticancer drugs and CCL20. The three-dimensional structure of CCL20 (PDB ID: 1M8A) was obtained from the Protein Data Bank (PDB) database (https://www.rcsb.org/), and candidate small-molecule compounds were obtained from the PubChem database (https://pubchem.ncbi.nlm.nih.gov/). Ligand and receptor structures were preprocessed using AutoDockTools, and molecular docking was performed with AutoDock Vina. The binding affinity of each compound to CCL20 was quantified by binding energy (kcal/mol); a binding energy below −5 kcal/mol was considered indicative of favorable interaction and relatively stable complex formation.

### 2.10. Single-Cell RNA-Seq Analysis and In Silico CCL20 Knockout Analysis (scTenifoldKnk)

Single-cell transcriptomic data were obtained from GSE145370, including ESCA tumor tissues and matched adjacent normal tissues. Using Seurat (version 5.3.0), Control and Disease samples underwent quality control filtering, LogNormalize normalization, selection of 2000 highly variable genes, dimensionality reduction, and batch-effect correction with Harmony, followed by graph-based clustering and t-SNE visualization (Figure S1). Cell–cell communication was analyzed using CellChat (version 1.6.1). To further characterize the CCL20-CCR6 axis, monocyte, macrophage, and T-cell populations were subsetted for secondary analysis and re-clustered using Seurat. The subsetted cells were re-normalized, re-scaled, and re-clustered based on 15 principal components with a resolution of 0.30 for monocytes and macrophages, and 20 principal components with a resolution of 0.35 for T cells. CellChat networks for the Control and Disease groups were then reconstructed to evaluate intercellular communication patterns, with a minimum cell-count threshold of 10 cells per subtype. Macrophage and monocyte subpopulations from the Disease group were subsequently subjected to scTenifoldKnk (version 1.0.1).

### 2.11. Pan-Cancer Data Acquisition and Functional Signature Analysis

Normal tissue expression and cell type-enhanced annotation of CCL20 were obtained from the Human Protein Atlas (https://www.proteinatlas.org/). Transcriptomic and clinical data from 33 TCGA cancer types were obtained from TCGA (https://www.cancer.gov/ccg/research/genome-sequencing/tcga, accessed on 1 February 2026) and used for pan-cancer analyses of CCL20, including expression distribution, overall survival, MSI, TMB, and correlations with immune-related gene panels. The prognostic value of CCL20 was evaluated by univariable Cox regression. To characterize context-dependent functional associations, CancerSEA-derived signatures representing 14 cancer-related phenotypes were obtained from CancerSEA (http://biocc.hrbmu.edu.cn/CancerSEA/, accessed on 1 February 2026), and curated gene sets for regulated cell-death programs were analyzed in parallel. For each cancer type, only tumor samples were retained according to TCGA sample-type barcodes (codes 01–09); duplicated genes were averaged, gene sets with fewer than five overlapping genes were excluded, and sample-level signature scores were calculated using GSVA in z-score mode followed by within-cancer normalization. Pearson correlation analysis between CCL20 expression and normalized signature scores was then performed within each cancer type and visualized as radar plots, forest plots, and heatmaps.

### 2.12. Tissue Microarray and Immunohistochemistry (IHC)

All immunohistochemical (IHC) analyses were performed using a commercially available tissue microarray (TMA; Catalog No. AF-EsoSur2201, AiFang Biological, Changsha, China). The TMA contained 160 cores, including 80 paired esophageal squamous cell carcinoma (ESCC) tissues and matched adjacent non-tumorous tissues. Clinicopathological characteristics and survival data were provided by the manufacturer. This TMA was used to evaluate CCL20 protein expression by IHC and to further assess its association with clinicopathological features and clinical outcomes (Table S4). This study was approved by the Ethics Committee of Hunan AiFang Biological (Approval No. HN20250401). All samples were collected after written informed consent had been obtained from the patients.

Briefly, TMA sections were deparaffinized and subjected to antigen retrieval using EDTA buffer. The sections were then incubated overnight at 4 °C with a primary antibody against CCL20 (Affinity Biosciences, Beijing, China; Catalog No. DF2238). After washing, the sections were incubated with a secondary antibody for 50 min, followed by visualization with 3,3′-diaminobenzidine (DAB). Finally, the sections were counterstained with hematoxylin. Whole-slide images were scanned using a PANNORAMIC digital slide scanner (LG-S80, Servicebio, Wuhan, China). Quantitative analysis was performed on the digital TMA images using the artificial intelligence-based digital pathology image analysis software Aipathwell (version 2.3.1) (Servicebio, China). The staining intensity and the proportion of positive cells were quantified, and the integrated optical density (IOD) of the positive regions in each image was measured. The average optical density (AOD) was calculated as IOD divided by the stained area. When more than one analyzable region was available for a given sample, the mean AOD value was used for subsequent statistical analyses.

### 2.13. Statistical Analysis

All statistical analyses were performed in R (version 4.4.2). Comparisons between two groups were conducted using the Wilcoxon rank-sum test, and correlation analyses were performed using Spearman correlation. Survival analyses were performed using Cox proportional hazards regression models, and HRs with 95% CIs were reported. For analyses involving multiple comparisons, FDR correction was performed using the Benjamini–Hochberg (BH) method. Unless otherwise specified, all tests were two-sided, and *p* < 0.05 was considered statistically significant.

## 3. Results

### 3.1. Differential Gene Expression in Esophageal Cancer

**Figure 1:**
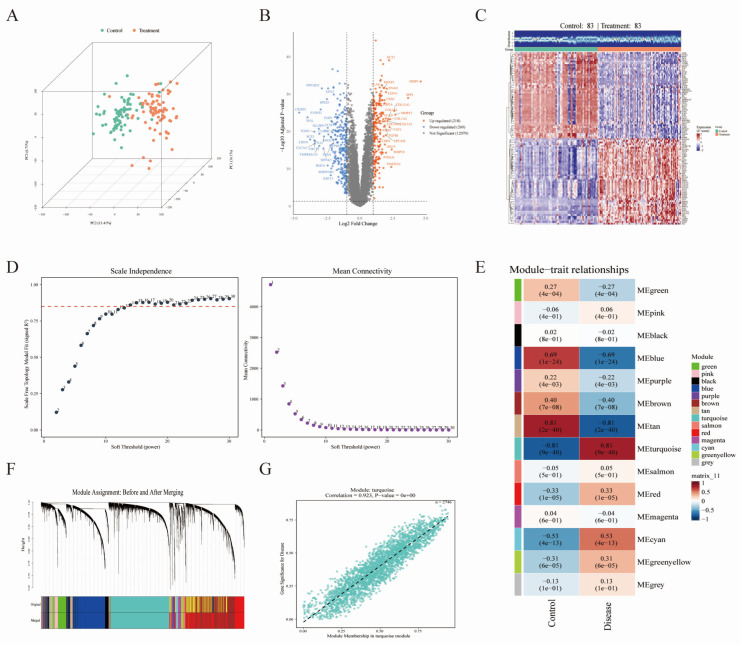
Differential expression and WGCNA in the merged GEO discovery cohort (GSE23400 and GSE38129). (**A**) Three-dimensional principal component analysis (PCA) showing the overall transcriptomic separation between control and tumor samples. (**B**) Volcano plot of differentially expressed genes (DEGs), including 218 upregulated genes and 260 downregulated genes. (**C**) Heatmap showing the expression patterns of DEGs across control and tumor samples. (**D**) Soft-threshold selection for WGCNA, showing the scale-free topology fit index and mean connectivity across different powers. The numbers 1–30 indicate the candidate soft-thresholding powers tested during WGCNA network construction. (**E**) Module–trait relationship heatmap showing the correlations between co-expression modules and phenotype. (**F**) Gene dendrogram and module assignment before and after module merging. Colors indicate different WGCNA co-expression modules identified by dynamic tree cutting and after module merging. (**G**) Scatter plot showing the correlation between module membership and gene significance in the turquoise module.

In the merged GEO discovery cohort (GSE23400 and GSE38129), using |log2FC| > 1 and adjusted FDR < 0.05 as the thresholds, a total of 478 DEGs were identified, including 218 upregulated and 260 downregulated genes (Figures S2 and 1B). Three-dimensional PCA showed clear separation between the two groups at the global transcriptomic level (PC1 = 16.1%, PC2 = 13.41%, PC3 = 6.71%), indicating marked overall transcriptional differences between the control and disease groups (Figure 1A). The heatmap generated from the DEGs showed distinct clustering of the control and disease groups based on expression patterns, with good within-group consistency across samples (Figure 1C).

### 3.2. Construction of the WGCNA Co-Expression Network

To further identify phenotype-associated key modules at the co-expression network level, we constructed a weighted gene co-expression network using WGCNA. Soft-threshold analysis indicated that the network at power = 14 adequately satisfied the scale-free topology criterion while maintaining a reasonable mean connectivity (Figure 1D). After module merging, 14 gene co-expression modules were identified, each represented by a different color (Figure 1F); among them, the MEturquoise module showed the strongest positive correlation with disease samples (r = 0.81, *p* = 9 × 10^−40^) (Figure 1E). In addition, module membership (MM) and gene significance (GS) were strongly correlated within the MEturquoise module (r = 0.923, *p* < 2.2 × 10^−16^), suggesting that hub genes in this module may play important roles in disease-related molecular mechanisms (Figure 1G).

### 3.3. Integrated Screening and Prognostic Evaluation Identified CCL20 as a Key Prognostic Biomarker

**Figure 2:**
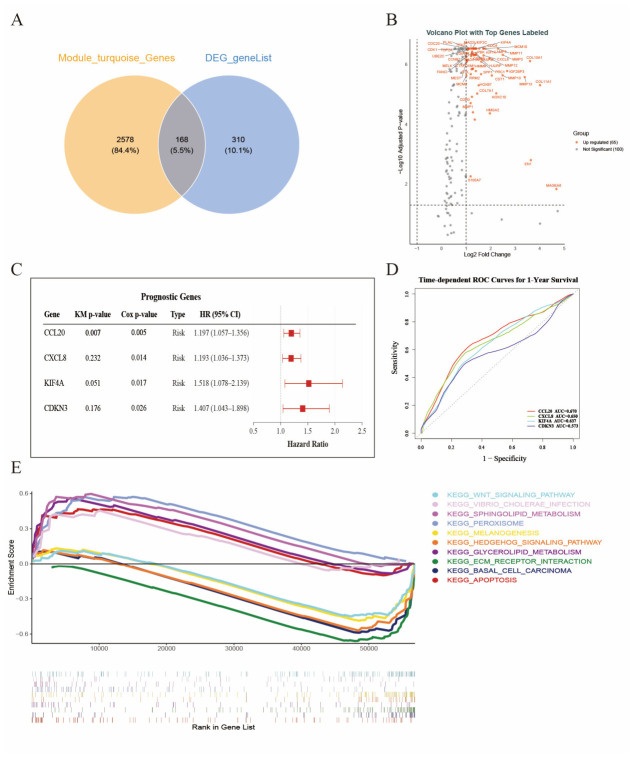
Candidate gene screening, external validation, and functional pathway analysis. (**A**) Venn diagram showing the overlap between genes in the key WGCNA turquoise module and DEGs. (**B**) Volcano plot of intersecting candidate genes in the TCGA-ESCA cohort, with selected significantly altered genes annotated. (**C**) Forest plot showing the top prognostic candidate genes identified by Kaplan–Meier and univariable Cox regression analyses. (**D**) Time-dependent ROC curves for 1-year survival prediction of prognostic candidate genes. (**E**) KEGG-GSEA plot based on CCL20 expression grouping, showing representative pathways associated with CCL20 (In panel (**B**), dotted lines indicate the predefined thresholds for fold change and adjusted *p* value. In panel (**C**), the vertical dotted line indicates the reference line of HR = 1. In panel (**D**), the diagonal dotted line indicates the reference line for random classification. In panel (**E**), the horizontal reference line indicates an enrichment score of 0.

We first intersected genes from the WGCNA key module (turquoise) with the DEGs, yielding 168 candidate genes (Figure 2A), indicating that these genes shared both co-expression module centrality and differential expression significance. To evaluate the robustness of these candidate genes in an independent cohort, we analyzed the TCGA esophageal cancer dataset. Among them, 65 genes remained significantly differentially expressed in TCGA (Figure 2B), indicating good cross-cohort stability of the candidate genes. We next performed Kaplan–Meier and univariable Cox regression analyses to evaluate their prognostic relevance. CCL20, CXCL8, KIF4A, and CDKN3 were identified as the top prognostic candidate genes, among which CCL20 showed the most stable significance in both analyses (KM *p* = 0.007, Cox *p* = 0.005, HR = 1.197, 95% CI: 1.057–1.356), whereas the other genes reached statistical significance mainly in Cox analysis but showed weaker performance in Kaplan–Meier analysis (Figure 2C). Further 1-year time-dependent ROC analysis showed that CCL20 had the highest AUC (0.670), compared with CXCL8 (0.650), KIF4A (0.637), and CDKN3 (0.573) (Figure 2D), supporting its superior overall prognostic discrimination. To explore potential biological mechanisms, KEGG-GSEA based on CCL20 expression indicated that several pathways related to tumor development and the immune microenvironment may be involved, including the WNT signaling pathway, Hedgehog signaling pathway, ECM–receptor interaction, and apoptosis (Figure 2E), providing clues to the potential biological role of CCL20.

### 3.4. Validation of CCL20 Expression in Pan-Cancer and Esophageal Cancer

**Figure 3:**
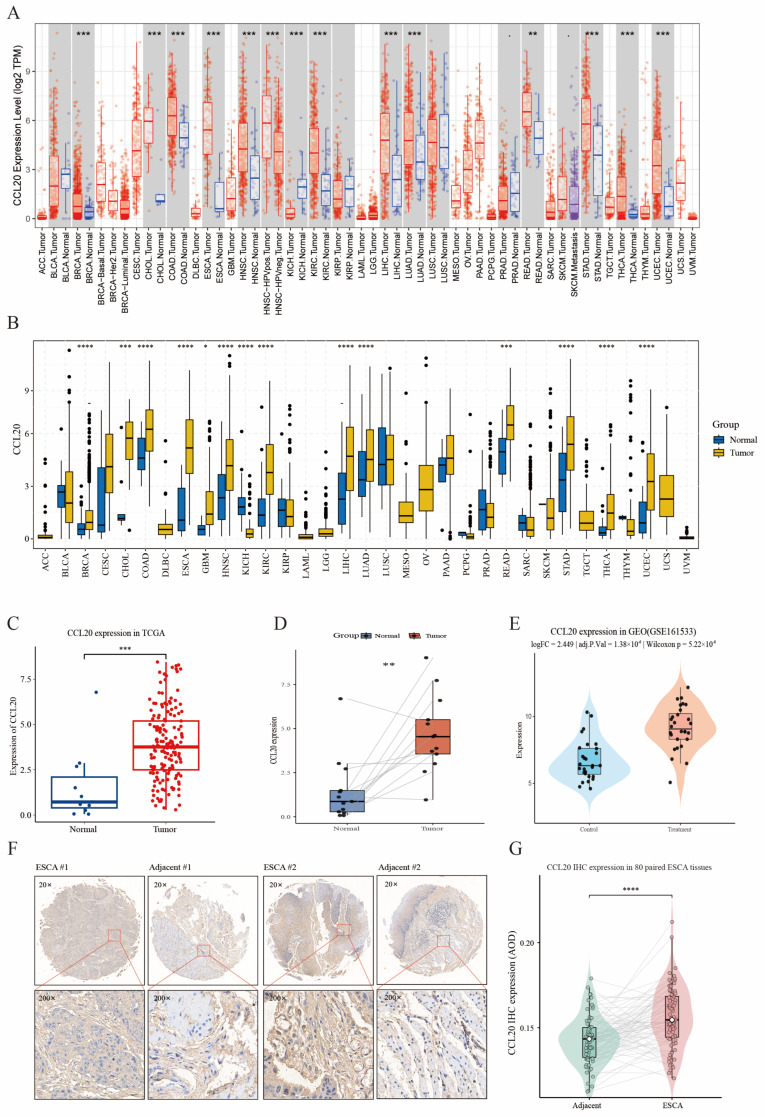
Validation of CCL20 expression across pan-cancer datasets, independent cohorts, and protein level. (**A**) Comparison of CCL20 expression between tumor and normal tissues in the integrated pan-cancer dataset. In the expression plots, red indicates tumor samples and blue indicates normal samples (**B**) Comparison of CCL20 expression between tumor and normal tissues across TCGA cancer types. (**C**) Unpaired expression comparison of CCL20 between normal and tumor samples in the TCGA-ESCA cohort. (**D**) Paired expression comparison of CCL20 in matched normal and tumor samples from the TCGA-ESCA cohort. (**E**) External validation of CCL20 expression in the GSE161533 cohort. (**F**) Representative immunohistochemical staining of CCL20 in ESCA tissues and matched adjacent tissues at 20× and 200× magnifications. #1 and #2 indicate representative paired samples from patient 1 and patient 2, respectively. (**G**) Quantitative analysis of CCL20 IHC expression in 80 paired ESCA and adjacent tissues (* *p* < 0.05, ** *p* < 0.01, *** *p* < 0.001, **** *p* < 0.0001).

To further validate the expression pattern of CCL20, we first performed pan-cancer analysis. Both the integrated dataset and TCGA pan-cancer analysis showed that CCL20 was aberrantly expressed across multiple tumor types and was significantly elevated in ESCA (Figure 3A,B). In the TCGA-ESCA cohort, CCL20 expression was significantly higher in unpaired tumor tissues than in normal tissues (logFC = 1.352, adjusted FDR = 4.85 × 10^−4^, Wilcoxon *p* = 4.41 × 10^−5^) (Figure 3C). This difference remained significant in 13 matched tumor–normal pairs (*p* = 0.0046) (Figure 3D). The external GSE161533 cohort yielded consistent results, showing significant upregulation of CCL20 in the case group (logFC = 2.449, adjusted *p* = 1.38 × 10^−6^, Wilcoxon *p* = 5.22 × 10^−6^) (Figure 3E and Figure S4). At the protein level, IHC showed stronger CCL20 staining in ESCA tissues than in matched adjacent tissues (Figure 3F). Quantitative analysis of 80 paired samples further confirmed significantly increased CCL20 protein expression in tumor tissues (paired Wilcoxon *p* = 1.49 × 10^−6^) (Figure 3G). Moreover, compared with existing literature-reported biomarkers in esophageal cancer, CCL20 showed the best overall diagnostic and prognostic performance, with the highest diagnostic AUC (0.839) and 1-year time-dependent AUC (0.667), and it was the only marker that remained significant in both log-rank (*p* = 0.00623) and Cox analyses (HR = 1.197, 95% CI: 1.057–1.356, *p* = 0.00454) (Table S3, Figure S5).

### 3.5. High CCL20 Expression Predicts an Unfavorable Prognosis in Patients with ESCA

**Figure 4:**
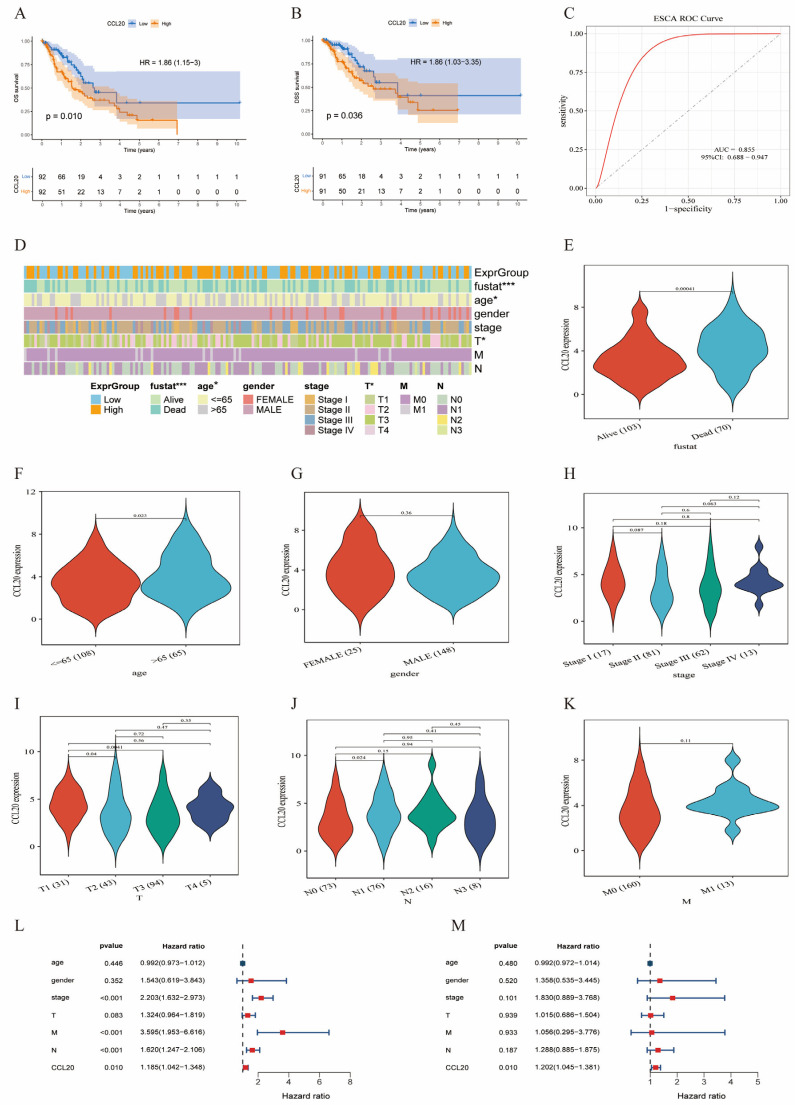
Association between CCL20 expression and clinical characteristics. (**A**) Kaplan–Meier curve of overall survival (OS) in patients with ESCA stratified by CCL20 expression. (**B**) Kaplan–Meier curve of disease-specific survival (DSS) in patients with ESCA stratified by CCL20 expression. (**C**) ROC curve evaluating the discriminatory performance of CCL20 for ESCA. In the ROC plot, the diagonal dotted line indicates the reference line for random classification. (**D**) Association between CCL20 expression groups and clinicopathological characteristics of patients with ESCA (including survival status, age, sex, clinical stage, T stage, N stage, and M stage). (**E**–**K**) Differences in CCL20 expression across clinical subgroups. (**L**) Univariable Cox regression analysis. (**M**) Multivariable Cox regression analysis. Statistical significance is indicated as follows: * *p* < 0.05 and *** *p* < 0.001.

In the TCGA-ESCA cohort, Kaplan–Meier analysis showed that patients with high CCL20 expression had significantly worse overall survival (OS) (Figure 4A), and disease-specific survival (DSS) was also reduced (Figure 4B). ROC analysis indicated that CCL20 had good discriminatory performance for ESCA (Figure 4C). Consistent findings were obtained in the tissue microarray cohort, where IHC-based CCL20 expression showed moderate diagnostic value (Figure S3B; AUC = 0.722, 95% CI: 0.642–0.802), and patients with high CCL20 protein expression had significantly shorter OS than those with low expression (Figure S3C; HR = 1.89, 95% CI: 1.02–3.52, *p* = 0.032).

### 3.6. Association of CCL20 with Clinicopathological Characteristics in the TCGA Cohort

Clinical correlation analysis showed that CCL20 expression differed across survival status (fustat) and age strata (Figure 4E,F, Table 1), whereas no clear difference was observed by sex (Figure 4G); CCL20 expression also showed stratified trends across stage and TNM subgroups (Figure 4H–K). Consistently, tissue microarray-based clinicopathological analysis showed that high CCL20 IHC expression was associated with shorter overall survival time and more advanced N stage (Table S4). Further Cox regression analysis showed that CCL20 was significantly associated with OS in the univariable model (HR = 1.185, *p* = 0.010; Figure 4L) and remained independently associated with prognosis after adjustment for clinical variables in the multivariable model (HR = 1.202, *p* = 0.010; Figure 4M), suggesting that CCL20 may serve as a potential independent adverse prognostic biomarker in ESCA. In addition, we developed and deployed an online interactive survival analysis platform based on Shiny (https://lokkey.shinyapps.io/km_shiny_online/, accessed on 12 February 2026), which allows users to input clinical information and automatically perform data preprocessing, grouping, Kaplan–Meier survival curve generation, log-rank testing, and univariable Cox regression analysis, and to dynamically estimate 1-, 3-, and 5-year survival probabilities for patients with esophageal cancer based on the input CCL20 expression level.

### 3.7. Association Between CCL20 Expression and Immune Infiltration

**Figure 5:**
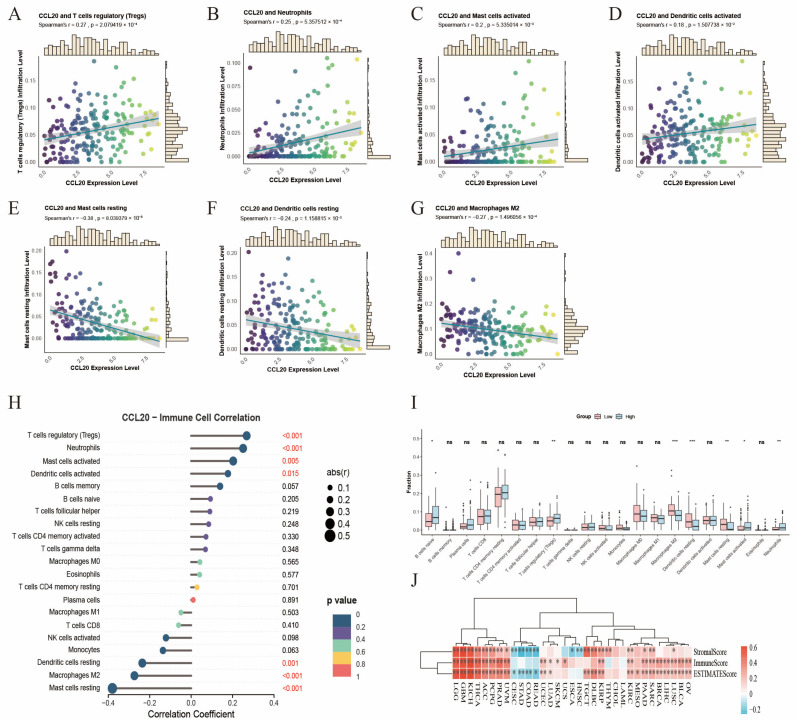
Association of CCL20 expression with immune infiltration and the tumor microenvironment. (**A**–**G**) Scatter plots showing the correlations between tumor-infiltrating immune cells and CCL20 expression. In the scatter plots, dots represent individual samples, the fitted line indicates the correlation trend between CCL20 expression and immune cell infiltration level, and the shaded area represents the 95% confidence interval. The color gradient of the dots indicates the relative CCL20 expression level. (**H**) Forest plot showing the correlations between CCL20 expression and tumor-infiltrating immune cells. (**I**) Comparison of immune cell infiltration proportions between the high- and low-CCL20-expression groups. (**J**) Heatmap showing differences in StromalScore, ImmuneScore, and ESTIMATE score between high- and low-CCL20-expression groups across multiple cancers. Statistical significance is indicated as follows: * *p* < 0.05, ** *p* < 0.01, and *** *p* < 0.001.

The CIBERSORT method was used to assess the association between CCL20 expression and immune cell infiltration. As shown in the figure, high CCL20 expression was positively correlated with the infiltration levels of regulatory T cells (Tregs), neutrophils, activated mast cells, and activated dendritic cells, but negatively correlated with resting mast cells, resting dendritic cells, and M2 macrophages (Figure 5A–H). Patients were further stratified into high- and low-CCL20 expression groups based on the median expression level for comparison of immune cell proportions; the high-expression group showed significant enrichment of Tregs, neutrophils, and multiple activated immune cell subsets, whereas the proportions of some resting phenotypes or other subsets were relatively reduced (Figure 5I). In addition, analysis of immune-related metrics showed that the high-CCL20-expression group had a significantly lower StromalScore in patients with ESCA, suggesting that CCL20 expression is associated with altered stromal characteristics rather than simple stromal enrichment (Figure 5J). Taken together, high CCL20 expression was associated with distinct immune infiltration patterns and reduced stromal scores, indicating that CCL20 may be involved in remodeling the immune and stromal components of the esophageal cancer tumor microenvironment.

### 3.8. CCL20 Expression as a Treatment-Related Prognostic Factor in ESCA

**Figure 6:**
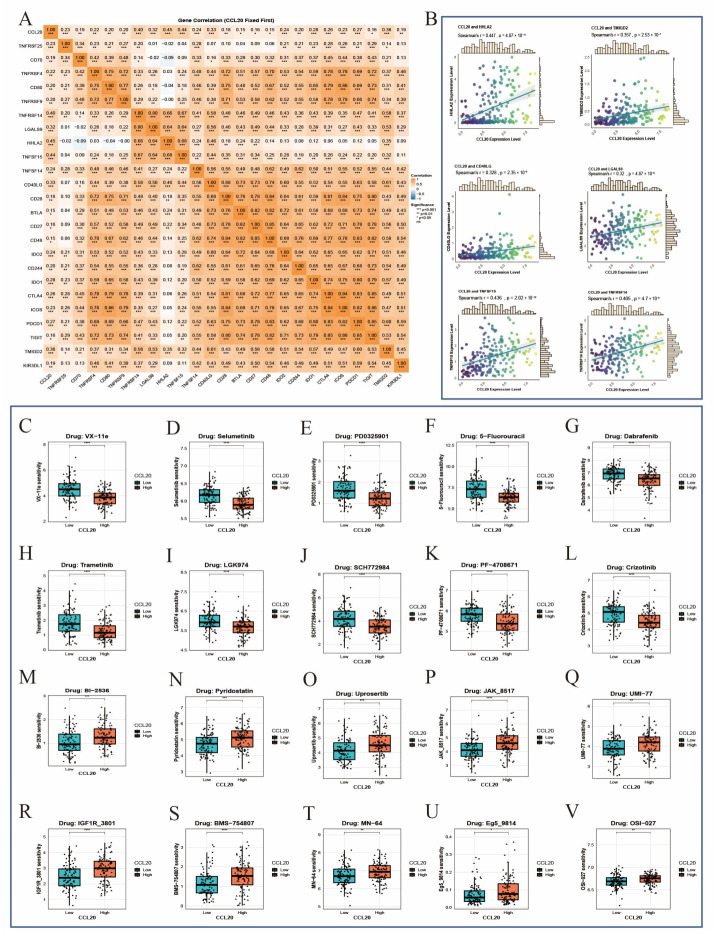
Correlation analysis of CCL20 with immune checkpoints and drug sensitivity. (**A**) Heatmap showing correlations between CCL20 and multiple immune checkpoint-related genes. (**B**) Scatter plots showing correlations between CCL20 expression and immune checkpoints, including HHLA2, TMIGD2, and CD40LG. In the scatter plots, dots represent individual samples, the fitted line indicates the correlation trend between CCL20 expression and immune cell infiltration level, and the shaded area represents the 95% confidence interval. The color gradient of the dots indicates the relative CCL20 expression level. (**C**–**V**) Drug sensitivity analysis in the high- and low-CCL20-expression subgroups. Statistical significance is indicated as follows: * *p* < 0.05, ** *p* < 0.01, *** *p* < 0.001, and **** *p* < 0.0001.

To explore the potential role of CCL20 in immunotherapy, we examined its associations with immune checkpoint molecules in patients with esophageal cancer. CCL20 expression showed relatively strong positive correlations with HHLA2, TMIGD2, CD40LG, LGALS9, TNFSF15, and TNFRSF14 and was also positively correlated with multiple other checkpoint molecules (Figure 6A,B). In addition, drug sensitivity analysis indicated that predicted responses to anticancer agents in patients with ESCA varied according to CCL20 expression level. In the high-CCL20-expression subgroup, the predicted IC50 values for VX-11e, Selumetinib, PD0325901, 5-Fluorouracil, Dabrafenib, Trametinib, LGK974, SCH772984, PF-4708671, and Crizotinib were significantly lower, suggesting greater sensitivity to these agents in patients with higher CCL20 expression (Figure 6C–L). Conversely, in the low-CCL20 expression subgroup, BI-2536, Pyridostatin, Uprosertib, JAK_8517, UMI-77, IGF1R_3801, BMS-754807, MN-64, Eg5_9814, and OSI-027 showed lower predicted IC50 values, suggesting that patients with lower CCL20 expression may be more sensitive to these drugs (Figure 6M–V). These findings suggest that CCL20 levels may influence the response of esophageal cancer to multiple targeted agents and chemotherapeutic drugs and may provide a potential reference for individualized treatment strategies.

### 3.9. Molecular Docking Analysis

**Figure 7:**
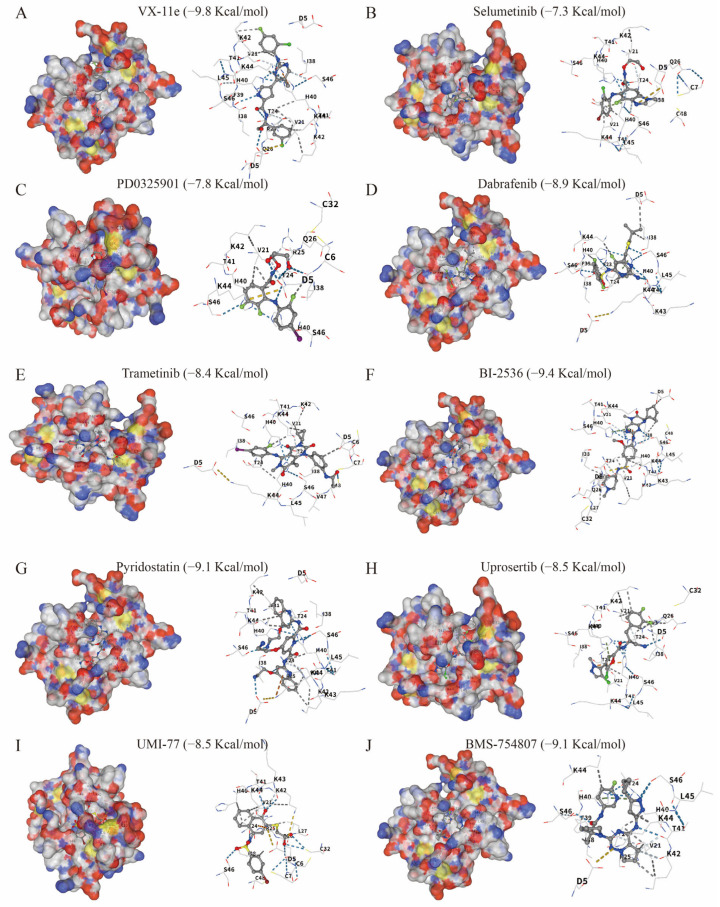
Molecular docking results of CCL20 with candidate small-molecule drugs. (**A**) VX-11e. (**B**) Selumetinib. (**C**) PD0325901. (**D**) Dabrafenib. (**E**) Trametinib. (**F**) BI-2536. (**G**) Pyridostatin. (**H**) Uprosertib. (**I**) UMI-77. (**J**) BMS-754807.

In the molecular docking analysis, all 10 selected candidate compounds showed favorable predicted binding to CCL20 (Figure 7). Among them, VX-11e exhibited the lowest predicted binding energy (−9.8 kcal/mol), followed by BMS-754807 and pyridostatin (both −9.1 kcal/mol), while Dabrafenib, Uprosertib, and UMI-77 showed binding energies of −8.9, −8.5, and −8.5 kcal/mol, respectively. Trametinib, PD0325901, and Selumetinib also demonstrated relatively low binding energies, ranging from −8.4 to −7.3 kcal/mol. Structural inspection suggested that the predicted ligand–protein interactions were mainly stabilized by hydrogen bonding and hydrophobic contacts involving residues such as K44, S46, H40, and L45. These results provide computational evidence that the selected compounds may have the capacity to interact with putative binding regions of CCL20, although the biological relevance of these interactions requires further experimental validation.

### 3.10. Validation of the CCL20–CCR6 Axis at the Single-Cell Transcriptomic Level and In Silico CCL20 Knockout Analysis

**Figure 8:**
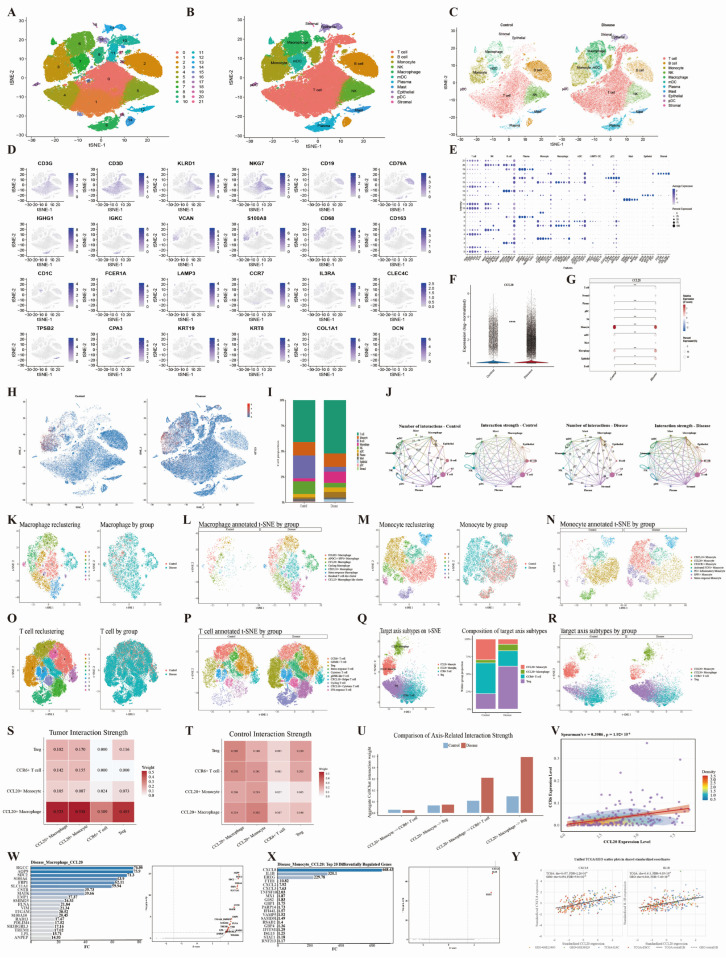
Validation of the CCL20–CCR6 axis at the single-cell transcriptomic level and in silico CCL20 knockout analysis. (**A**,**B**) t-SNE plots showing all cell clusters and annotated major cell types. (**C**) t-SNE plots showing cell distribution in the Control and Disease groups. (**D**,**E**) Feature and dot plots of representative marker genes for cell type identification. In the t-SNE plots, dots represent individual cells, and colors indicate cell types. (**F**–**H**) Overall, cell-type-specific and spatial expression patterns of CCL20 in the Control and Disease groups. (**I**) Stacked bar plot showing the composition of major cell populations in the two groups. (**J**) CellChat-inferred cell–cell communication networks showing changes in interaction number and strength between the Control and Disease groups. In the CellChat network plots, lines indicate inferred intercellular communication links, and line thickness represents interaction strength. (**K**–**P**) Reclustering and annotation of macrophage, monocyte, and T-cell subtypes, with group-stratified distributions. (**Q**,**R**) Global projection, composition, and group-specific distribution of four axis-related subpopulations, including CCL20+ monocytes, CCL20+ macrophages, CCR6+ T cells, and Tregs. (**S**,**T**) Heatmaps showing aggregate CellChat interaction strength among the four axis-related subpopulations in the Disease and Control groups. (**U**) Comparison of aggregate CellChat interaction strength for selected myeloid-to-lymphoid signaling directions between the two groups. (**V**) Scatter plot showing the correlation between CCL20 and CCR6 expression in TCGA-ESCA. (**W**,**X**) Top 20 differentially regulated genes and corresponding volcano plots after in silico CCL20 knockout in Disease-group macrophages and monocytes. (**Y**) Scatter plots showing the correlations of CCL20 with representative downstream targets in TCGA and GEO cohorts. Statistical significance is indicated as follows: * *p* < 0.05, ** *p* < 0.01, *** *p* < 0.001, and **** *p* < 0.0001.

Based on single-cell transcriptomic data from esophageal cancer, we first constructed and annotated the cellular atlas. t-SNE clustering identified 11 major cell populations, including T cells, B cells, NK cells, monocytes, macrophages, mDCs, mast cells, plasma cells, epithelial cells, pDCs, and stromal cells (Figure 8A,B). The overall cell distribution was broadly similar between the Control and Disease groups, but the Disease group showed relatively higher proportions of macrophages, T cells, and plasma cells, whereas the proportions of B cells and NK cells were reduced (Figure 8C,I). Feature plots and dot plots of canonical marker genes further supported the above cell type annotations (Figure 8D,E). On this basis, we evaluated the single-cell expression pattern of CCL20. Overall, CCL20 transcript levels were elevated in the Disease group (Figure 8F), with expression mainly concentrated in monocytes and macrophages and relatively low expression in other immune cell types (Figure 8G,H). CellChat-based comparison of cell–cell communication networks showed that both the number and strength of overall interactions were increased in the Disease group relative to the Control group (Figure 8J).

To further refine the putative CCL20-CCR6 immune axis, we performed secondary reclustering of the macrophage, monocyte, and T-cell compartments. This analysis identified several key subpopulations, including CCL20+ macrophages, CCL20+ monocytes, CCR6+ T cells, and Tregs (Figure 8K–R). When these four axis-related subpopulations were projected back onto the global t-SNE space, the Disease group showed a clear redistribution toward Tregs and CCL20+ macrophages, whereas CCL20+ monocytes and CCR6+ T cells were relatively reduced (Figure 8Q,R). Focused CellChat analysis of these axis-related populations showed that the most prominent disease-associated gains involved communication from CCL20+ macrophages to Tregs and CCR6+ T cells, whereas monocyte-derived signaling changed more modestly (Figure 8S–U). Consistent with this finding, patient-level bulk analysis in TCGA-ESCA showed a significant positive correlation between CCL20 and CCR6 expression (Figure 8V). In addition, the high-CCL20/high-CCR6 subgroup in TCGA-ESCA displayed a more immunoregulatory phenotype, characterized by increased Treg infiltration and higher expression of multiple Treg/exhaustion-associated markers, including FOXP3, IL2RA, TIGIT, PDCD1, CTLA4, and LGALS9 (Figure S6B–D). Supplementary single-cell analysis further supported complementary localization of CCL20 and CCR6 across myeloid and lymphoid/dendritic compartments (Figure S6E).

We further performed in silico CCL20 knockout in macrophages and monocytes from the Disease group using scTenifoldKnk. The results showed widespread transcriptional perturbations in both cell types after knockout; in macrophages, several key genes related to inflammatory responses, immune chemotaxis, and extracellular matrix remodeling, such as RGCC, AQP9, SDC2, and S100A6, were markedly dysregulated. In monocytes, in silico CCL20 knockout was associated with clear dysregulation of inflammatory mediators, including CXCL8, IL1B, and EREG (Figure 8W,X). Further validation in independent TCGA and GEO cohorts showed that CXCL8 and IL1B remained positively correlated with CCL20 at the bulk-tissue level (Figure 8Y and Figure S7). These findings suggest that CCL20 is aberrantly activated mainly in myeloid cells within the esophageal cancer microenvironment and may strengthen communication between CCL20+ macrophages and Tregs/CCR6+ T cells through the CCL20-CCR6 axis, while reshaping inflammatory transcriptional programs, thereby potentially contributing to immune microenvironment dysregulation and tumor progression in esophageal cancer.

### 3.11. Integrated Pan-Cancer Analysis of Prognostic, Immune-Related, and Functional Characteristics of CCL20

**Figure 9:**
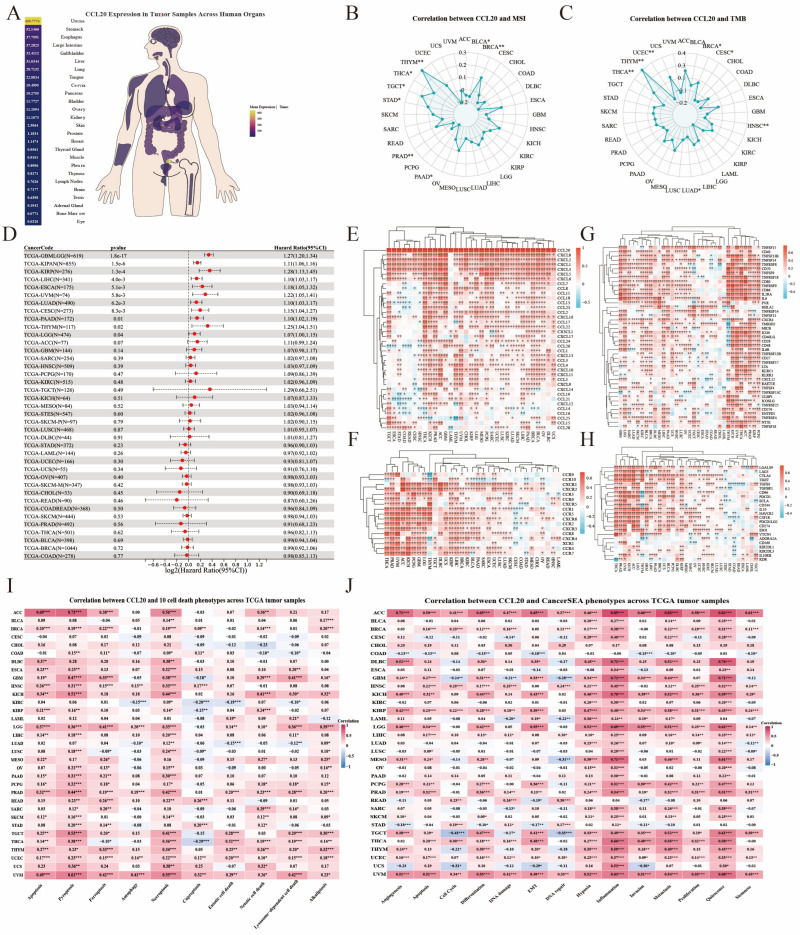
Pan-cancer functional features and immune-related analyses of CCL20. (**A**) Organ-level distribution of CCL20 expression across pan-cancer tumor samples. (**B**,**C**) Correlations of CCL20 with MSI and TMB across cancer types. (**D**) Association between CCL20 expression and overall survival across pan-cancer cohorts. Red dots represent hazard ratio estimates, and horizontal lines represent the corresponding 95% confidence intervals. (**E**–**H**) Co-expression patterns of CCL20 with chemokines, chemokine receptors, immune regulatory molecules, and immune checkpoint genes across cancer types. (**I**) Correlation between CCL20 and 10 cell-death phenotypes across TCGA tumor samples. (**J**) Correlation between CCL20 and CancerSEA phenotypes across TCGA tumor samples. Statistical significance is indicated as follows: * *p* < 0.05, ** *p* < 0.01, and *** *p* < 0.001.

In pan-cancer cohorts, organ-level visualization of tumor samples showed that CCL20 expression was highest in the uterus and was also relatively elevated in several digestive and mucosal organs, including the stomach, esophagus, large intestine, gallbladder, and liver (Figure 9A). Further analysis revealed marked heterogeneity in the correlations of CCL20 with MSI and TMB across cancer types, although significant positive correlations were observed in several tumors, particularly THCA and THYM (Figure 9B,C). Survival analysis showed that high CCL20 expression was significantly associated with poor overall survival in multiple independent cohorts, including GBMLGG, KIPAN, KIRP, LIHC, ESCA, UVM, LUAD, CESC, PAAD, THYM, and LGG (Figure 9D), supporting CCL20 as a potential pan-cancer adverse prognostic biomarker.

We next systematically examined the immune-related co-expression patterns of CCL20. Clustered heatmaps showed that CCL20 was broadly positively correlated with multiple CCL/CXCL chemokines (Figure 9E) and chemokine receptors of the CCR/CXCR families (Figure 9F). CCL20 was also highly co-expressed with a range of immune regulatory molecules, including TNF/TNFRSF family members, CD40, CD70, CD80, IL2RA, and ENTPD1 (Figure 9G), as well as canonical immune checkpoint genes such as CTLA4, PDCD1, CD274, TIGIT, LAG3, and HAVCR2 (Figure 9H). In addition, cell-death analysis showed that CCL20 was most consistently and strongly positively associated with pyroptosis and necroptosis across tumor types, followed by apoptosis and ferroptosis (Figure 9I). These associations were particularly evident in tumors such as ACC, UVM, LGG, GBM, and KICH, whereas correlations with other cell-death programs, including cuproptosis and parthanatos, were comparatively weaker and more heterogeneous. CancerSEA-derived functional phenotype analysis further showed that inflammation was the most prominent correlate of CCL20 across cancers, followed by quiescence, metastasis, and hypoxia (Figure 9J). Positive associations with malignant phenotypes, including EMT, invasion, proliferation, and angiogenesis, were also observed in multiple tumor types, although the strength and direction of these correlations varied substantially across cancers. Collectively, these findings indicate that CCL20 is broadly linked to immune-regulatory pathways, inflammatory cell-death programs, and context-dependent malignant phenotypes across cancers and may participate in tumor immune microenvironment reprogramming by reshaping chemokine axes and immune checkpoint networks.

## 4. Discussion

Using an integrative multi-omics framework, this study systematically characterized the expression pattern, clinical relevance, immune associations, and translational implications of CCL20 in esophageal cancer and across cancers. Overall, CCL20 was aberrantly upregulated in multiple solid tumors and was associated with adverse prognosis in several cancer types. In ESCA, elevated CCL20 expression was consistently supported at both the transcriptomic and protein levels and was associated with poorer survival, enhanced infiltration of immunoregulatory cell populations, increased expression of immune checkpoint molecules, and distinct predicted drug-response patterns. Integrative analyses combining single-cell transcriptomics with in silico perturbation further suggested that the CCL20–CCR6 axis may contribute to myeloid-associated inflammatory signaling and immune microenvironment remodeling, thereby highlighting CCL20 as a biologically relevant candidate biomarker and a putative therapeutic target in ESCA.

At the pan-cancer level, CCL20 was elevated in multiple tumor types, including BRCA, COAD, HNSC, KIRC, LIHC, LUAD, and STAD, and was associated with worse overall survival in several cohorts, including ESCA, LIHC, and LUAD. These findings are consistent with recent pan-cancer studies of inflammation–aging-related genes, in which CCL20, together with CXCL8 and CCL24, contributed to a high-risk signature associated with tumor progression and poor prognosis [19]. Previous studies also linked high CCL20 expression to EMT activation, enhanced inflammatory responses, and TNF signaling, and to poorer survival and less favorable immunotherapy responses in cancers such as lung adenocarcinoma [20]. In line with these findings, KEGG-based GSEA in ESCA identified significant enrichment of WNT signaling, Hedgehog signaling, ECM–receptor interaction, and apoptosis-related pathways in the CCL20 expression-stratified analysis, suggesting that CCL20 may be associated with tumor progression and microenvironment remodeling. This observation is supported by recent single-cell evidence from APC-mutant colorectal cancer, in which CCL20 was identified as a key immune microenvironment regulator and was reported to be upregulated through APC inactivation/WNT/MYC-related signaling [15]. CancerSEA analysis further showed that CCL20 was positively associated with inflammation-related functions in most cancers and, in NSCLC, LUAD, HNSCC, and BRCA, with EMT, invasion, and metastasis; in some tumors, it was also associated with hypoxia and proliferation, suggesting that CCL20 may link inflammatory signaling to malignant progression. Elevated serum CCL20 has also been associated with relapse after immunotherapy and primary resistance to immune checkpoint inhibitors in melanoma, further supporting a role for CCL20 in treatment response regulation [21]. In esophageal cancer, prior studies have shown that promoter demethylation can drive CCL20 upregulation and is associated with ESCA progression and immune dysregulation; single-cell evidence further suggests that increased CCL20 expression is accompanied by CCR6+ Th17/Treg imbalance, indicating a possible early immune perturbation signal during ESCA development [18]. Other studies reported that transcription factors such as Eomes can directly upregulate CCL20 and promote the proliferation and metastasis of esophageal cancer cells [17]. The importance of myeloid remodeling in ESCC is further supported by evidence that tumor-intrinsic FOXO1 promotes tumor progression by increasing M2 macrophage infiltration, indicating that macrophage-skewed immune remodeling is clinically relevant in esophageal cancer [12]. Tumor-associated microorganisms, such as Fusobacterium nucleatum, can also significantly induce CCL20 expression [22]. Clinically, serum CCL20 is elevated in patients with esophageal cancer and correlates with disease occurrence and distant metastasis [23], and combined CCL4/CCL20 expression patterns may improve prognostic stratification [24,25]. Against this background, our GEO–TCGA integration and WGCNA analyses further support CCL20 as a stable ESCA-associated module gene, whereas the TCGA cohort together with tissue microarray-based validation suggests that CCL20 has potential diagnostic and prognostic value.

Our integrated bulk, single-cell, and subgroup analyses support a model in which the CCL20–CCR6 axis contributes to immunosuppressive remodeling of the ESCA tumor microenvironment. In the bulk transcriptomic analysis, high CCL20 expression was associated with increased Treg infiltration and upregulation of multiple inhibitory immune molecules. This pattern was further reinforced by subgroup analysis showing that the CCL20-high/CCR6-high subgroup exhibited higher Treg fractions, increased expression of FOXP3 and CTLA4, and more prominent stromal features, collectively indicating a more immunoregulatory microenvironment. At the single-cell level, CCL20 was predominantly expressed in monocytes/macrophages, whereas CCR6 was enriched in lymphoid and dendritic compartments, and CellChat analysis showed that one of the most prominent disease-associated gains in intercellular communication was directed from CCL20+ macrophages toward Tregs and CCR6+ T cells. Taken together, these findings suggest that the increased communication strength observed at the single-cell level may be reflected at the tissue level by the accumulation of CCR6-positive immunoregulatory lymphocytes, particularly Tregs, thereby linking myeloid-derived CCL20 signaling to the bulk-level immune infiltration pattern. This interpretation is biologically plausible and consistent with previous studies showing that the CCL20–CCR6 axis promotes Treg recruitment and immunosuppressive remodeling in esophageal cancer and other solid tumors [11,12,13,17,26,27,28,29]. In addition to its chemotactic role, recent evidence suggests that CCR6–CCL20 signaling may also enhance the functional fitness of tumor-infiltrating Tregs [11,13], suggesting that targeting the CCL20-CCR6 axis offers a promising therapeutic strategy. Accordingly, our results support the view that CCL20-high ESCA does not simply represent an immune-inflamed state, but rather an “inflamed but suppressed” phenotype characterized by coexistence of immune activation signals and inhibitory regulation. Nevertheless, because CCR6 is also expressed in other immune subsets and the consequences of this axis are context-dependent across tumor types [30], the precise cellular and functional effects of CCL20 in ESCA still require further mechanistic validation.

To further explore the regulatory role of CCL20, we applied scTenifoldKnk, an in silico single-cell gene knockout method based on single-cell gene regulatory networks and manifold alignment [31]. In macrophages, CCL20 knockout was associated with perturbation of transcriptional networks involving AQP9, S100A6, RGCC, and SDC2. Given the reported roles of AQP9 in macrophage metabolic adaptation and pro-angiogenic phenotypes [32], and of S100A6 in proliferation, cytoskeletal remodeling, and migration [33,34], these findings suggest that CCL20 may be involved in regulating myeloid metabolic reprogramming and migration-related programs. In monocytes, CCL20 knockout also altered CXCL8, IL1B, and EREG, including CXCL8, a key chemokine involved in the recruitment of myeloid-derived suppressor cells and pro-tumor neutrophils [35,36]. Together, these findings support the hypothesis that myeloid-derived CCL20 may function as an important node linking innate inflammatory programs and adaptive immune imbalance.

From a translational perspective, the druggability of the CCL20–CCR6 axis is more likely to reside in biologic neutralization of the ligand or pharmacologic blockade of the receptor than in direct small-molecule inhibition of secreted CCL20. This interpretation is consistent with current preclinical and early translational evidence. In humans, a monoclonal antibody against CCL20 selectively inhibited recruitment of CCR6-positive cells in an experimental blister model [37]. In cancer-related preclinical settings, anti-CCL20 treatment suppressed osteolytic breast cancer bone metastasis [38], and inhibition of the CCR6–CCL20 axis reduced intratumoral Treg recruitment and enhanced radiotherapy response in head and neck squamous cell carcinoma [28]. In parallel, receptor-directed strategies have also advanced, including an antagonistic anti-CCR6 antibody that reduced CCL20-induced chemotaxis and IL-17 expression [39], first-in-class small-molecule CCR6 inhibitors [40], and PF-07054894 [41], a potent CCR6 antagonist that has entered clinical evaluation. Against this background, the favorable docking energies observed for VX-11e and related compounds in our study should be regarded as exploratory and hypothesis-generating, rather than as evidence that these agents directly neutralize extracellular CCL20.

Biologically, the predicted association between high-CCL20 tumors and increased sensitivity to VX-11e, selumetinib, trametinib, and PD0325901 is more likely to reflect coupling with MAPK/ERK signaling than direct disruption of the ligand–receptor interaction itself. VX-11e is an ERK2 inhibitor, and published studies support bidirectional crosstalk between MAPK/ERK signaling and the CCL20–CCR6 axis: oncogenic EGFR/Ras activity can induce CCL20 production [42], whereas CCL20/CCR6 stimulation can activate ERK-dependent programs and promote tumor-cell proliferation and migration [43,44,45]. Therefore, the predicted drug-response profile in the high-CCL20 subgroup is more plausibly interpreted as a marker of MAPK/ERK-associated biological dependence upstream or downstream of the CCL20–CCR6 axis, rather than as proof of direct pharmacologic targeting of CCL20 itself. Under this framework, MEK/ERK inhibitors may still be of translational interest in high-CCL20 ESCA, but their potential activity is more likely to arise from indirect modulation of CCL20-associated signaling networks and should be validated experimentally.

This study has several limitations. First, the immune infiltration, microenvironmental, and drug-response analyses were inferred computationally and should not be interpreted as direct biological or clinical effects; in particular, GDSC2-based predictions are trained largely on cancer cell-line models. Second, the single-cell analysis was performed in a relatively small and heterogeneous cohort, which may affect the robustness of the inferred cell–cell communication landscape. Third, the virtual knockout results remain model-dependent and non-causal and therefore provide hypothesis-generating rather than definitive mechanistic evidence. Finally, although molecular docking and pharmacogenomic prediction support the potential therapeutic relevance of the CCL20 axis, these observations still require biochemical and functional validation.

In summary, this study identifies CCL20 as an inflammation-related chemokine with potential relevance to prognosis, immune remodeling, and treatment response in ESCA and across cancers. Our findings implicate the CCL20–CCR6 axis in myeloid-associated immune communication and immunosuppressive microenvironment remodeling, while drug-response prediction and molecular docking provide preliminary support for its therapeutic relevance. Further mechanistic and functional studies are needed to define the biological and translational significance of this axis in ESCA.

## 5. Conclusions

This study systematically evaluated the biological and clinical relevance of CCL20 in esophageal cancer and across cancers through an integrative multi-omics framework. Our findings indicate that CCL20 is aberrantly upregulated in ESCA and multiple other solid tumors and is associated with adverse prognosis, immune-related features, and distinct predicted therapeutic response patterns. In ESCA, the combined transcriptomic and single-cell evidence further implicates the CCL20–CCR6 axis in immunoregulatory signaling and tumor microenvironmental remodeling. Overall, these results highlight CCL20 as a biologically relevant candidate biomarker with potential diagnostic, prognostic, and therapeutic relevance in ESCA and provide a foundation for further mechanistic and translational investigation.

## Figures and Tables

**Table 1:** Clinical characteristics and expression analysis of CCL20.

Feature	Group	Mean	SD	Number	*p* Value	Test
fustat	Alive	3.270609	1.785715	103	0.000405	*t*-test
	Dead	4.324486	1.937494	70		
age	≤65	3.432172	1.802392	108	0.022600	*t*-test
	>65	4.137109	2.026367	65		
gender	FEMALE	4.080879	2.263457	25	0.355000	*t*-test
	MALE	3.632194	1.850350	148		
stage	Stage I	4.256312	1.709279	17	0.125000	Kruskal
	Stage II	3.502869	1.902188	81		
	Stage III	3.654693	2.041783	62		
	Stage IV	4.377387	1.459120	13		
T	T1	4.421642	1.554186	31	0.034700	Kruskal
	T2	3.685190	2.158533	43		
	T3	3.444057	1.889538	94		
	T4	4.062244	1.360028	5		
M	M0	3.641754	1.940152	160	0.109000	*t*-test
	M1	4.377387	1.459120	13		
N	N0	3.345794	1.875278	73	0.118000	Kruskal
	N1	3.987831	1.929976	76		
	N2	4.054849	1.853059	16		
	N3	3.423873	2.014767	8		

## Data Availability

The datasets used and/or analyzed during the current study are available from the corresponding author on reasonable request.

## Supplementary Materials

The following supporting information can be downloaded at: https://www.mdpi.com/article/10.3390/biomedicines14051062/s1, Tables S1–S5, including differential expression results, WGCNA module genes, biomarker comparison, IHC clinicopathological analysis, and Cox regression results; Figures S1–S7, including single-cell quality control, batch-effect correction, IHC validation, external validation, biomarker comparison, CCL20–CCR6 axis analysis, and cross-cohort validation; and Supplementary Methods.

## Abbreviations

adrenocortical carcinoma (ACC), bladder urothelial carcinoma (BLCA), breast invasive carcinoma (BRCA), cervical squamous cell carcinoma and endocervical adenocarcinoma (CESC), cholangiocarcinoma (CHOL), colon adenocarcinoma (COAD), lymphoid neoplasm diffuse large B-cell lymphoma (DLBC), esophageal carcinoma (ESCA), glioblastoma multiforme (GBM), head and neck squamous cell carcinoma (HNSC), kidney chromophobe (KICH), kidney renal clear cell carcinoma (KIRC), kidney renal papillary cell carcinoma (KIRP), acute myeloid leukemia (LAML), brain lower-grade glioma (LGG), liver hepatocellular carcinoma (LIHC), lung adenocarcinoma (LUAD), lung squamous cell carcinoma (LUSC), mesothelioma (MESO), ovarian serous cystadenocarcinoma (OV), pancreatic adenocarcinoma (PAAD), pheochromocytoma and paraganglioma (PCPG), prostate adenocarcinoma (PRAD), rectum adenocarcinoma (READ), sarcoma (SARC), skin cutaneous melanoma (SKCM), stomach adenocarcinoma (STAD), testicular germ cell tumors (TGCT), thyroid carcinoma (THCA), thymoma (THYM), uterine corpus endometrial carcinoma (UCEC), uterine carcinosarcoma (UCS), and uveal melanoma (UVM).
