# Supplementary material for: Integrative Multi-Omics and Pan-Cancer Analyses Identify CCL20 as a Prognostic Biomarker with Therapeutic Relevance in Esophageal Cancer
Source: Biomedicines. 2026 May 7;14(5):1062. doi: 10.3390/biomedicines14051062 (PMC13204176; doi:10.3390/biomedicines14051062)
Supplement: Supplementary file 1 [file biomedicines-14-01062-s001.zip › Supplementary Materials.pdf]

Supplementary Materials

Table S1. Top 20 differentially expressed genes in the merged GEO discovery cohort. Page 2

Table S2. Top 20 representative genes in the turquoise module ranked by GS (Disease). Page 3

Table S3. Comparison of the diagnostic and prognostic performance of CCL20 with existing literature-reported biomarkers in esophageal cancer. Page 4

Table S4. Association between CCL20 IHC expression and clinicopathological characteristics in patients with ESCA. Page 5

Table S5. Univariate Cox regression analysis of candidate genes for overall survival in patients with esophageal cancer. Page 6

Figure S1. Quality control and preliminary annotation of the single-cell RNA-seq dataset. Page 8

Figure S2. Evaluation of batch effect correction in the merged GSE23400 and GSE38129 cohorts. Page 9

Figure S3. Immunohistochemical expression of CCL20 and its diagnostic and prognostic value in ESCA. Page 10

Figure S4. External validation of CCL20 expression patterns and diagnostic performance in the GSE161533 cohort. Page 11

Figure S5. Comparative evaluation of CCL20 and previously reported ESCA-related biomarkers in the TCGA cohort. Page 12

Figure S6. Subgroup features and single-cell expression pattern of the CCL20/CCR6 axis. Page 13

Figure S7. Cross-cohort bulk co-expression validation of CCL20 virtual-knockout-associated genes. Page 14

Supplementary Methods Page 15

Table S1. Top 20 differentially expressed genes in the merged GEO discovery cohort.

Rank	Gene	logFC	SE	AveExpr	t	*P* value	Adjusted *P* value	B
1	CBX3	1.192	0.056	9.173	21.206	3.653E-49	4.584E-45	101.191
2	CKS1B	1.556	0.082	9.125	19.015	1.423E-43	8.925E-40	88.491
3	ECT2	2.200	0.116	7.111	18.939	2.242E-43	9.376E-40	88.042
4	RFC4	1.756	0.095	7.818	18.510	2.995E-42	9.397E-39	85.481
5	KAT2B	-2.087	0.116	7.631	-17.956	8.834E-41	2.217E-37	82.137
6	UBL3	-1.688	0.095	8.512	-17.729	3.543E-40	7.410E-37	80.764
7	KIF4A	1.646	0.095	6.840	17.242	7.218E-39	1.294E-35	77.784
8	KIF14	1.571	0.093	6.072	16.822	9.891E-38	1.551E-34	75.195
9	MCM2	1.540	0.092	8.297	16.702	2.095E-37	2.921E-34	74.452
10	MMP1	4.603	0.277	7.200	16.623	3.438E-37	4.314E-34	73.962
11	GPD1L	-1.552	0.095	8.602	-16.408	1.322E-36	1.508E-33	72.630
12	CRYL1	-1.141	0.070	7.660	-16.375	1.630E-36	1.705E-33	72.422
13	COL1A1	1.665	0.103	8.015	16.200	4.902E-36	4.393E-33	71.333
14	NUCB2	-2.012	0.126	8.689	-16.010	1.628E-35	1.362E-32	70.145
15	LAPTM4B	1.498	0.094	9.774	15.958	2.265E-35	1.776E-32	69.819
16	MFAP2	1.843	0.116	7.824	15.917	2.930E-35	2.163E-32	69.564
17	MGLL	-2.007	0.126	9.280	-15.906	3.143E-35	2.174E-32	69.494
18	PLAU	1.803	0.114	7.520	15.872	3.899E-35	2.446E-32	69.281
19	PPP1R3C	-2.893	0.183	8.900	-15.830	5.094E-35	3.044E-32	69.016
20	GPX3	-2.421	0.153	9.783	-15.817	5.523E-35	3.150E-32	68.936

Notes:

1. Genes were ranked by adjusted *P* value in ascending order, with absolute logFC used as the secondary ranking key.
2. logFC represents the log2 fold change for tumor versus normal tissue.
3. SE represents the standard error of the estimated logFC.
4. AveExpr represents the average log2 expression value across samples in the merged GEO discovery cohort.
5. t represents the moderated t-statistic from the differential expression analysis, reflecting the ratio of the estimated logFC to its standard error after empirical Bayes moderation.
6. *P* value and Adjusted *P* value were derived from differential expression analysis; Adjusted *P* value was corrected using the Benjamini-Hochberg method.
7. B represents the log-odds that a gene is differentially expressed.

Table S2. **Top 20 representative genes in the turquoise module ranked by GS (Disease).**

Rank	Gene	Module	GS (Disease)	GS *P* value (Disease)	GS (Control)	GS *P* value (Control)	MM (turquoise)	MM *P* value	logFC	AveExpr	t	*P* value	Adjusted *P* value	B
1	CBX3	turquoise	0.855	1.127E-48	-0.855	1.127E-48	0.894	4.342E-59	1.192	9.173	21.206	3.653E-49	4.584E-45	101.191
2	CKS1B	turquoise	0.828	4.657E-43	-0.828	4.657E-43	0.924	2.502E-70	1.556	9.125	19.015	1.423E-43	8.925E-40	88.491
3	ECT2	turquoise	0.827	8.035E-43	-0.827	8.035E-43	0.928	5.287E-72	2.200	7.111	18.939	2.242E-43	9.376E-40	88.042
4	RFC4	turquoise	0.821	9.888E-42	-0.821	9.888E-42	0.906	4.172E-63	1.756	7.818	18.510	2.995E-42	9.397E-39	85.481
5	KIF4A	turquoise	0.801	2.150E-38	-0.801	2.150E-38	0.942	7.700E-80	1.646	6.840	17.242	7.218E-39	1.294E-35	77.784
6	KIF14	turquoise	0.794	2.829E-37	-0.794	2.829E-37	0.933	1.940E-74	1.571	6.072	16.822	9.891E-38	1.551E-34	75.195
7	MCM2	turquoise	0.792	5.912E-37	-0.792	5.912E-37	0.905	6.657E-63	1.540	8.297	16.702	2.095E-37	2.921E-34	74.452
8	MMP1	turquoise	0.790	1.090E-36	-0.790	1.090E-36	0.760	1.521E-32	4.603	7.200	16.623	3.438E-37	4.314E-34	73.962
9	LAPTM4B	turquoise	0.778	6.037E-35	-0.778	6.037E-35	0.786	4.312E-36	1.498	9.774	15.958	2.265E-35	1.776E-32	69.819
10	PLAU	turquoise	0.776	1.075E-34	-0.776	1.075E-34	0.774	2.214E-34	1.803	7.520	15.872	3.899E-35	2.446E-32	69.281
11	MCM6	turquoise	0.775	1.751E-34	-0.775	1.751E-34	0.818	3.742E-41	1.182	8.974	15.776	7.174E-35	3.810E-32	68.678
12	SNAI2	turquoise	0.774	2.031E-34	-0.774	2.031E-34	0.783	1.057E-35	2.031	8.234	15.774	7.287E-35	3.810E-32	68.662
13	PLXNA1	turquoise	0.774	2.127E-34	-0.774	2.127E-34	0.772	4.477E-34	0.799	8.074	15.707	1.110E-34	5.569E-32	68.246
14	FOXM1	turquoise	0.769	1.036E-33	-0.769	1.036E-33	0.901	2.372E-61	1.607	7.131	15.508	3.945E-34	1.904E-31	66.990
15	CEP55	turquoise	0.767	1.783E-33	-0.767	1.783E-33	0.930	5.891E-73	1.970	7.131	15.427	6.603E-34	3.069E-31	66.481
16	ACTL6A	turquoise	0.767	1.863E-33	-0.767	1.863E-33	0.890	1.051E-57	1.241	8.204	15.404	7.659E-34	3.432E-31	66.334
17	SNRPG	turquoise	0.766	2.391E-33	-0.766	2.391E-33	0.893	9.942E-59	0.888	10.222	15.339	1.159E-33	5.015E-31	65.924
18	AURKA	turquoise	0.766	3.068E-33	-0.766	3.068E-33	0.924	3.590E-70	0.973	6.436	15.308	1.409E-33	5.773E-31	65.730
19	HMGB3	turquoise	0.763	6.000E-33	-0.763	6.000E-33	0.852	4.621E-48	1.523	8.099	15.228	2.358E-33	8.702E-31	65.221
20	NEK2	turquoise	0.760	1.523E-32	-0.760	1.523E-32	0.923	9.756E-70	0.993	5.821	15.057	6.996E-33	2.251E-30	64.144

Notes:

1. Genes were ranked by GS (Disease) in descending order, with MM (turquoise) used as the secondary ranking key.

2. GS (Gene Significance) indicates the correlation between individual gene expression and the clinical trait (Disease or Control).

3. MM (Module Membership) indicates the correlation between individual gene expression and the eigengene of the turquoise module.

4. logFC represents log2 fold change for tumor versus normal tissue; positive values indicate upregulation in tumor tissue and negative values indicate downregulation.

5. AveExpr represents the average log2 expression value across samples in the merged GEO discovery cohort.

6. *P* value and Adjusted *P* value were derived from differential expression analysis; Adjusted P value was corrected using the Benjamini-Hochberg method.

7. B represents the log-odds that a gene is differentially expressed.

8. The turquoise module was the module most strongly correlated with disease status in WGCNA analysis.

Table S3. Comparison of the diagnostic and prognostic performance of CCL20 with existing literature-reported biomarkers in esophageal cancer.

Gene	Diagnostic_AUC	Log-rank *P* value	HR	Lower 95% CI	Upper 95% CI	Cox_*P* value	1-year time-dependent AUC
CCL20	0.839	0.00623	1.197	1.057	1.356	0.00454	0.667
CEACAM5	0.546	0.0249	1.055	0.978	1.137	0.166	0.557
KRT19	0.623	0.464	1.036	0.943	1.139	0.462	0.493
SERPINB3	0.678	0.56	0.978	0.897	1.067	0.617	0.405
SERPINB4	0.702	0.995	1	0.908	1.101	0.999	0.444
TP63	0.695	0.14	0.962	0.889	1.041	0.337	0.437
KRT7	0.63	0.106	1.036	0.938	1.146	0.484	0.607
MUC5AC	0.746	0.459	0.994	0.914	1.081	0.882	0.522
MUC6	0.798	0.348	1.013	0.926	1.109	0.773	0.556
CDX2	0.663	0.465	1.146	0.995	1.319	0.0579	0.608
AGR2	0.605	0.391	1.017	0.946	1.094	0.644	0.534

Table S4. Association between CCL20 IHC expression and clinicopathological characteristics in patients with ESCA.

Variable	Low (n = 57)[1]	High (n = 23)[1]	*P* value[2]
**Age (years)**	62.00 (55.00, 68.00)	63.00 (54.00, 66.00)	0.479
**Overall survival (months)**	40.00 (19.00, 48.00)	24.00 (15.00, 41.00)	0.032
**Sex**			0.314
Female	5 (8.8%)	0 (0.0%)	
Male	52 (91.2%)	23 (100.0%)	
**Differentiation**			0.173
Grade 1	4 (7.0%)	1 (4.3%)	
Grade 2	6 (10.5%)	2 (8.7%)	
Grade 3	20 (35.1%)	10 (43.5%)	
Grade 4	22 (38.6%)	4 (17.4%)	
Grade 5	5 (8.8%)	6 (26.1%)	
**Clinical stage**			0.318
Stage I	6 (10.5%)	0 (0.0%)	
Stage II	18 (31.6%)	6 (26.1%)	
Stage III	29 (50.9%)	16 (69.6%)	
Stage IV	4 (7.0%)	1 (4.3%)	
**T stage**			0.306
T1	2 (3.5%)	0 (0.0%)	
T2	14 (24.6%)	2 (8.7%)	
T3	34 (59.6%)	17 (73.9%)	
T4	7 (12.3%)	4 (17.4%)	
T1	2 (3.5%)	0 (0.0%)	
**N stage**			0.034
N0	26 (45.6%)	6 (26.1%)	
N1	18 (31.6%)	5 (21.7%)	
N2	9 (15.8%)	11 (47.8%)	
N3	4 (7.0%)	1 (4.3%)	
**M stage**			>0.999
M0	54 (94.7%)	22 (95.7%)	
M1	3 (5.3%)	1 (4.3%)	
**Vital status**			0.137
Alive	29 (50.9%)	7 (30.4%)	
Dead	28 (49.1%)	16 (69.6%)	

Notes: The optimal cutoff value for tumor CCL20 AOD was determined using the surv_cutpoint function in the survminer package based on maximally selected rank statistics, with minprop = 0.20. Patients with tumor CCL20 AOD > 0.1661 were assigned to the high-expression group, and those with AOD ≤ 0.1661 were assigned to the low-expression group. Continuous variables are presented as median (IQR), and categorical variables are presented as n (%). P values were calculated using the

Mann–Whitney U test for continuous variables and Fisher's exact test for categorical variables.

[1]Median (Q1, Q3); n (%)

[2]Wilcoxon rank sum test; Fisher's exact test

Table S5. Univariate Cox regression analysis of candidate genes for overall survival in patients with esophageal cancer.

Gene	Log-rank *P* value	Coef (B)	SE	HR	95% CI	Cox *P*-value
**CCL20**	**0.007**	0.180	0.063	1.197	1.057-1.356	**0.005**
CXCL8	0.232	0.176	0.072	1.193	1.036-1.373	0.014
KIF4A	0.051	0.418	0.175	1.518	1.078-2.139	0.017
CDKN3	0.176	0.341	0.153	1.407	1.043-1.898	0.026
MMP12	0.037	0.130	0.069	1.139	0.994-1.305	0.060
RRM2	0.393	0.269	0.165	1.309	0.947-1.809	0.103
SNX10	0.874	0.200	0.137	1.221	0.933-1.598	0.145
COL10A1	0.612	-0.106	0.078	0.899	0.772-1.048	0.173
IGF2BP3	0.442	0.162	0.120	1.176	0.930-1.486	0.176
HMMR	0.264	0.202	0.156	1.223	0.901-1.660	0.196
GINS1	0.105	0.216	0.168	1.241	0.893-1.725	0.198
CDK1	0.726	0.218	0.171	1.243	0.890-1.736	0.202
CDH3	0.135	-0.090	0.073	0.914	0.793-1.054	0.218
CCNB2	0.601	0.202	0.172	1.224	0.875-1.714	0.238
EN1	0.606	-0.105	0.091	0.900	0.753-1.077	0.251
RAD51AP1	0.099	0.182	0.159	1.200	0.879-1.638	0.252
MEST	0.059	0.132	0.122	1.141	0.898-1.451	0.280
SPP1	0.327	0.055	0.053	1.057	0.952-1.173	0.301
KIF20A	0.640	0.147	0.147	1.158	0.868-1.545	0.317
KIF23	0.468	0.171	0.177	1.186	0.838-1.678	0.335
TPX2	0.138	0.154	0.166	1.166	0.843-1.613	0.353
PLAU	0.669	0.084	0.092	1.087	0.908-1.302	0.363
STIL	0.985	0.175	0.196	1.192	0.811-1.751	0.372
GINS2	0.327	0.130	0.149	1.139	0.850-1.527	0.383
MELK	0.910	0.140	0.166	1.150	0.831-1.592	0.398
MMP1	0.378	-0.053	0.064	0.948	0.836-1.075	0.407
CXCL1	0.951	0.060	0.072	1.062	0.921-1.224	0.409
TTK	0.816	0.136	0.174	1.146	0.815-1.612	0.433
AURKB	0.597	0.107	0.154	1.113	0.823-1.507	0.486
MMP11	0.718	-0.047	0.068	0.954	0.836-1.090	0.490
NDC80	0.856	0.091	0.148	1.095	0.819-1.464	0.541
HJURP	0.663	0.102	0.171	1.108	0.792-1.548	0.550
COL7A1	0.305	-0.042	0.075	0.959	0.828-1.110	0.571
KIF18B	0.650	0.094	0.170	1.099	0.787-1.535	0.580
TRIP13	0.876	-0.072	0.133	0.931	0.718-1.207	0.589
MCM2	0.893	-0.063	0.128	0.939	0.731-1.208	0.626
COL11A1	0.412	-0.040	0.087	0.961	0.809-1.140	0.644

FANCI	0.431	0.080	0.187	1.084	0.752-1.562	0.667
LAMP3	0.505	-0.040	0.104	0.961	0.784-1.178	0.701
DLGAP5	0.311	0.063	0.165	1.065	0.770-1.472	0.704
BUB1B	0.234	0.064	0.171	1.066	0.762-1.491	0.710
MMP13	0.354	-0.027	0.073	0.974	0.844-1.123	0.714
DTL	0.803	0.056	0.154	1.057	0.782-1.429	0.717
MMP3	0.901	0.023	0.066	1.023	0.900-1.164	0.725
ASPM	0.891	0.055	0.159	1.057	0.774-1.442	0.729
TOP2A	0.252	0.030	0.093	1.031	0.859-1.237	0.743
APOBEC3B	0.975	-0.041	0.125	0.960	0.752-1.226	0.745
KIF2C	0.971	0.054	0.167	1.056	0.761-1.464	0.746
PRC1	0.314	0.189	0.651	1.208	0.337-4.329	0.771
PBK	0.244	0.041	0.148	1.042	0.779-1.394	0.781
FOXM1	0.244	-0.036	0.138	0.965	0.736-1.265	0.797
MAD2L1	0.962	-0.042	0.167	0.959	0.691-1.331	0.804
MMP10	0.723	0.016	0.070	1.016	0.886-1.165	0.822
S100A7	0.669	-0.006	0.029	0.994	0.939-1.052	0.835
KIF14	0.601	-0.035	0.174	0.965	0.686-1.357	0.839
HOXC10	0.100	0.020	0.104	1.020	0.831-1.252	0.848
CST1	0.951	0.009	0.055	1.009	0.906-1.123	0.871
MAGEA6	0.957	-0.007	0.058	0.993	0.887-1.112	0.902
CDC6	0.178	0.013	0.116	1.013	0.806-1.273	0.911
MCM10	0.413	0.018	0.177	1.018	0.719-1.442	0.919
CDC20	0.607	-0.013	0.149	0.988	0.738-1.322	0.933
CEP55	0.806	-0.007	0.155	0.993	0.733-1.345	0.966
HMGA2	0.777	0.002	0.106	1.002	0.814-1.234	0.985
HOXB7	0.834	-0.001	0.130	0.999	0.774-1.290	0.994

Figure S1. Quality control and preliminary annotation of the single-cell RNA-seq dataset.

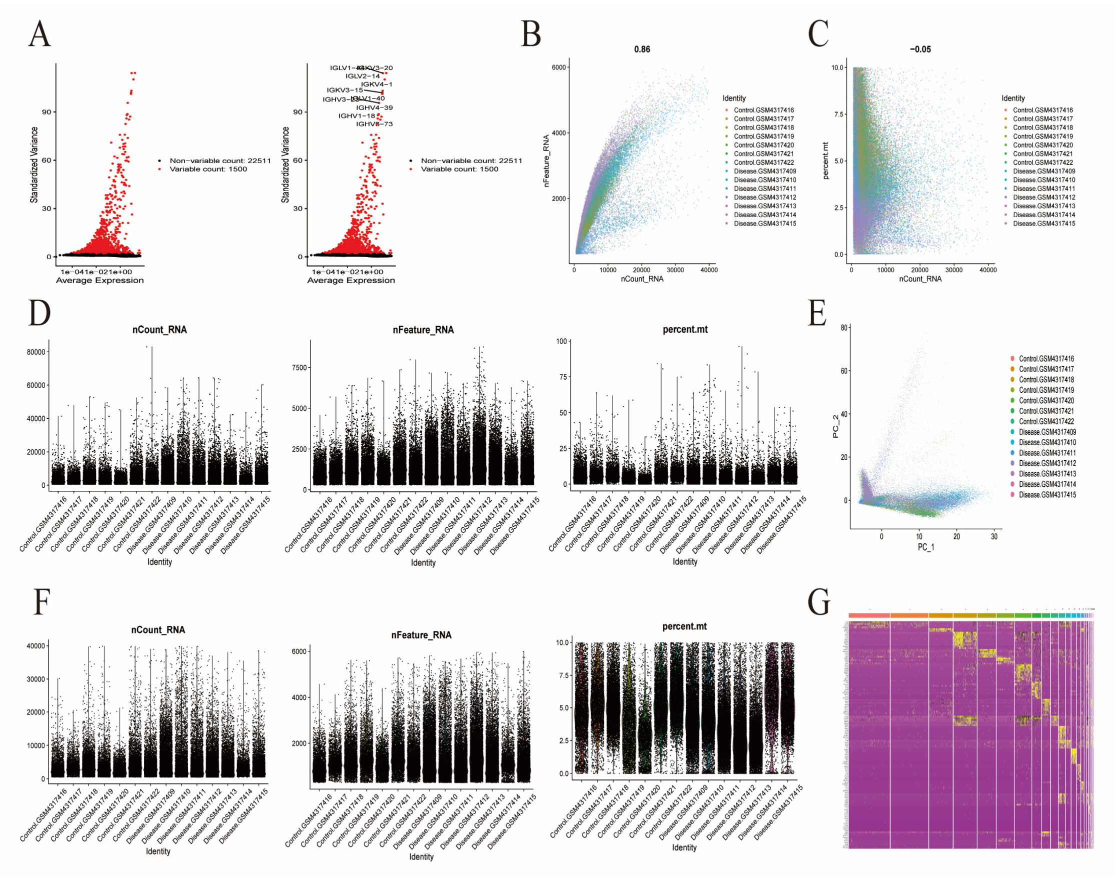

A: Scatter plot of highly variable genes, showing the relationship between mean expression and standardized variance.

B–C: Quality-control scatter plots: B, relationship between nCount_RNA and nFeature_RNA; C, relationship between nCount_RNA and mitochondrial gene percentage (percent.mt).

D: Distribution of nCount_RNA, nFeature_RNA, and percent.mt across samples before quality filtering.

E: PCA plot colored by sample, used to evaluate inter-sample variation and potential batch effects.

F: Distribution of nCount_RNA, nFeature_RNA, and percent.mt across samples after quality filtering.

G: Heatmap of representative marker genes for clustered cells (clusters 0–21), used for subsequent cell type annotation.

Figure S2. Evaluation of batch effect correction in the merged GSE23400 and GSE38129 cohorts.

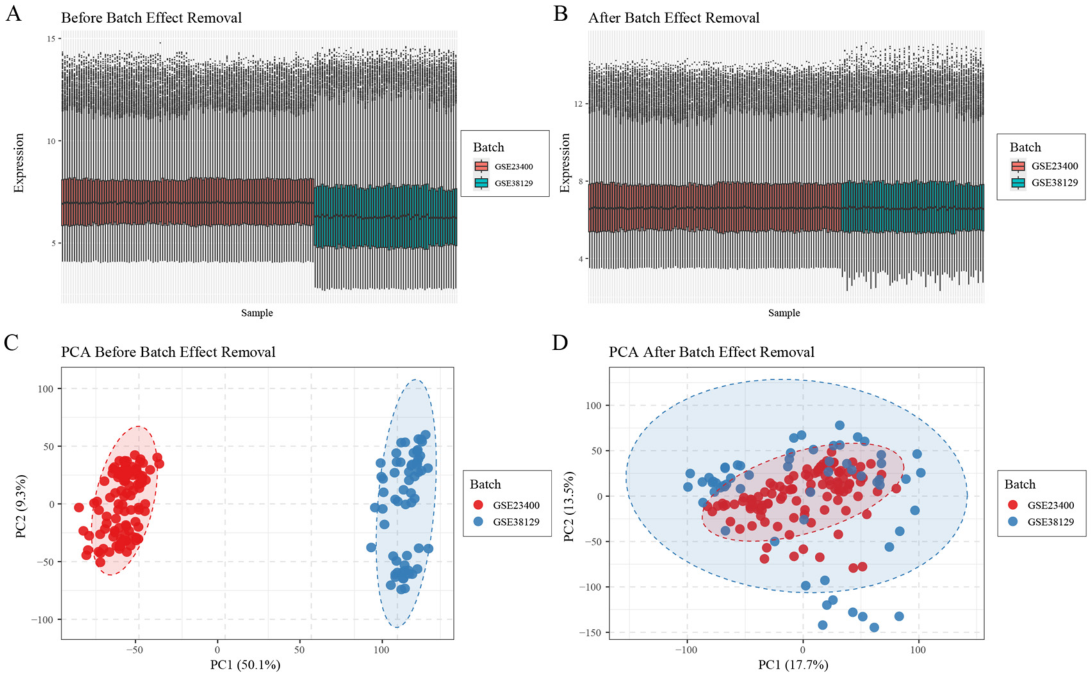

(A) Boxplots of gene expression distributions before batch effect removal.

(B) Boxplots of gene expression distributions after batch effect removal.

(C) PCA plot before batch effect removal.

(D) PCA plot after batch effect removal.

Figure S3. Immunohistochemical expression of CCL20 and its diagnostic and prognostic value in ESCA.

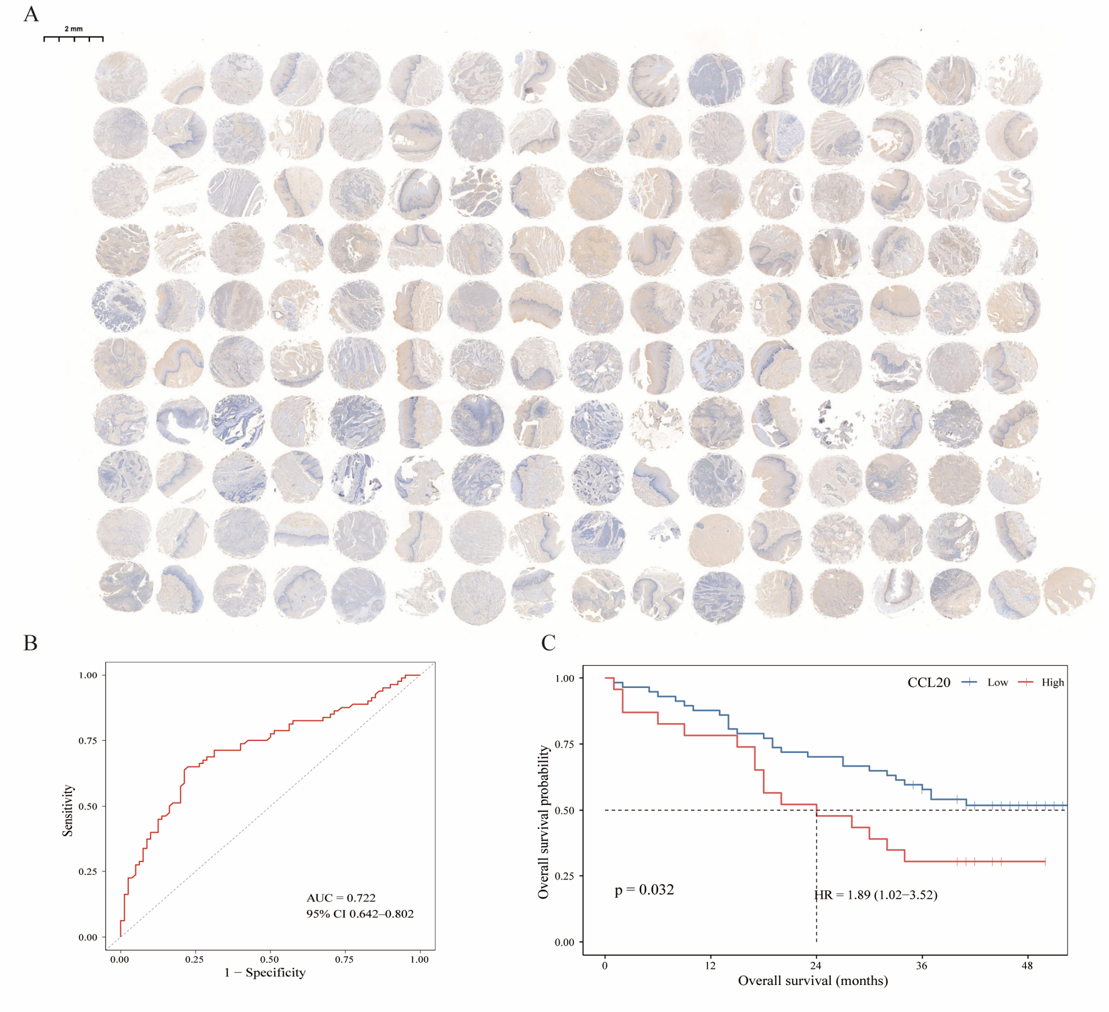

(A) Immunohistochemical staining of CCL20 in a tissue microarray comprising 80 paired ESCA tumor tissues and matched adjacent normal tissues.

(B) ROC curve showing the diagnostic value of CCL20 expression in ESCA. The AUC was 0.722 (95% CI, 0.642–0.802).

(C) Kaplan–Meier curve for overall survival in patients with ESCA stratified into high- and low-CCL20 expression groups using the optimal cutoff value. High CCL20 expression was associated with poorer overall survival (HR = 1.89, 95% CI: 1.02–3.52; log-rank $p = 0.032$).

Figure S4. External validation of CCL20 expression patterns and diagnostic performance in the GSE161533 cohort.

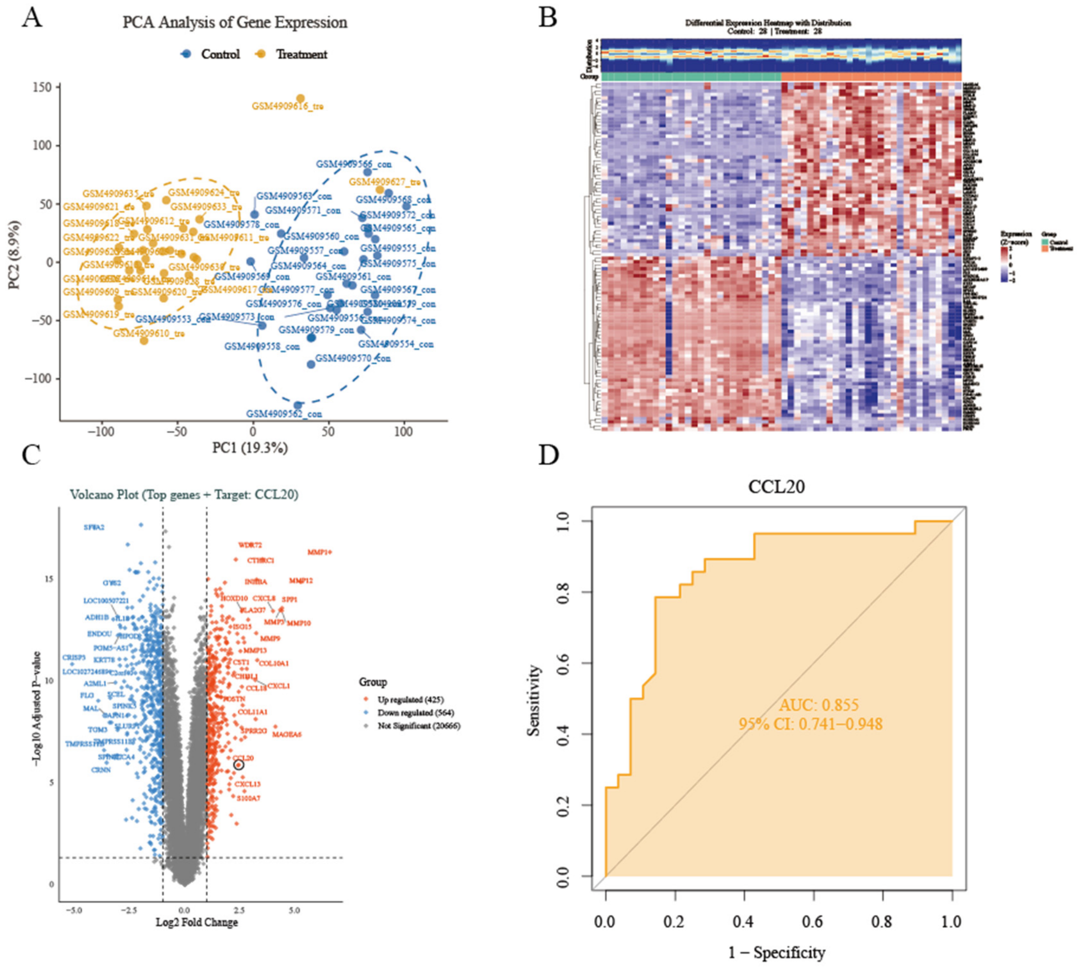

(A) PCA plot of samples in the control and treatment groups.

(B) Heatmap of differentially expressed genes between the control and treatment groups.

(C) Volcano plot showing differentially expressed genes, with CCL20 highlighted.

(D) ROC curve of CCL20 for distinguishing treatment samples from control samples (AUC = 0.855, 95% CI: 0.741-0.948).

Figure S5. Comparative evaluation of CCL20 and previously reported ESCA-related biomarkers in the TCGA cohort.

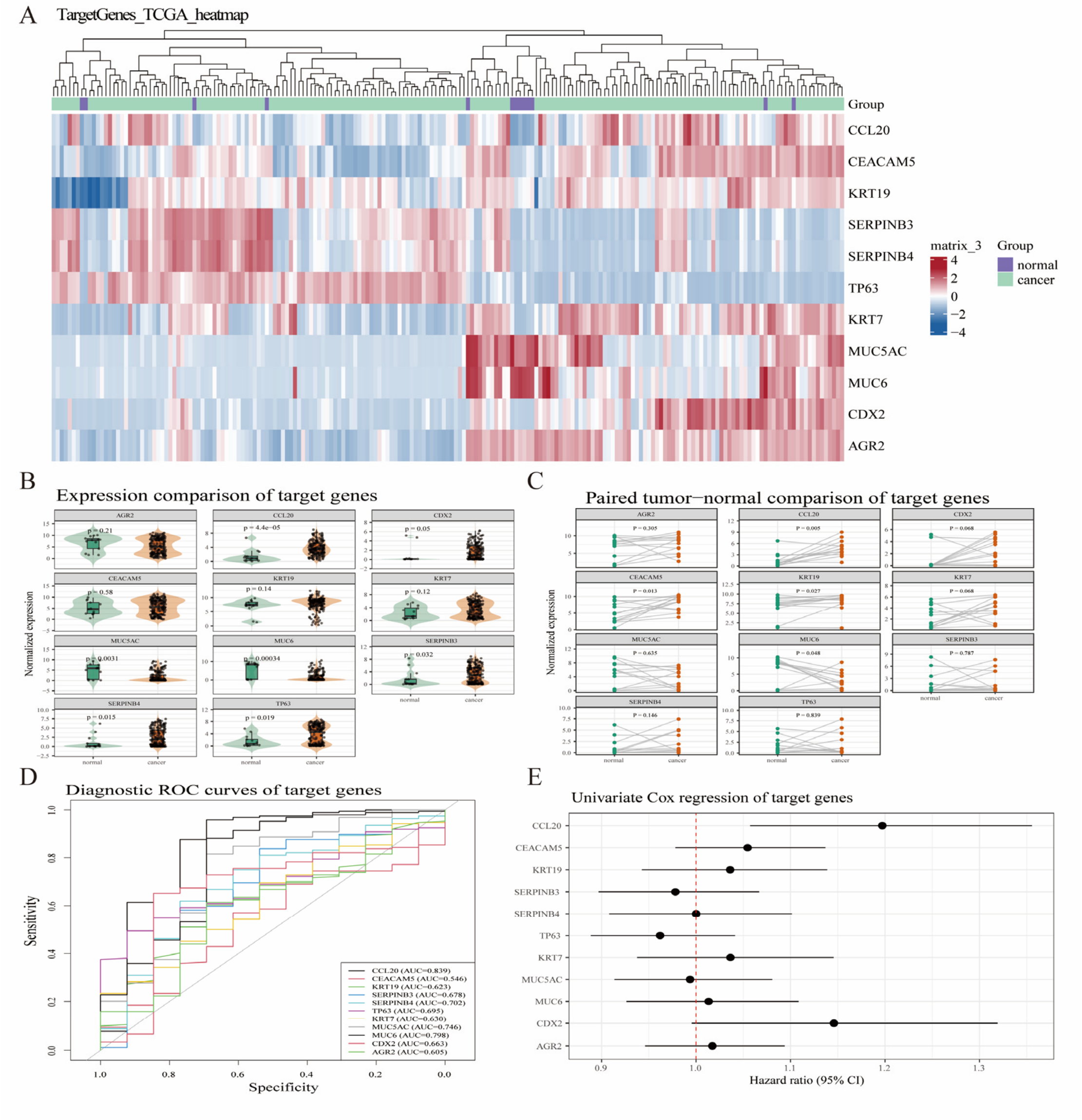

(A) Heatmap showing the expression patterns of CCL20 and previously reported ESCA-related biomarkers in tumor and normal samples from the TCGA cohort.

(B) Comparison of the expression levels of CCL20 and candidate biomarkers between tumor and normal tissues.

(C) Paired comparison of gene expression between tumor and matched adjacent normal tissues for CCL20 and the comparison biomarkers.

(D) Receiver operating characteristic (ROC) curves comparing the diagnostic performance of CCL20 with other reported ESCA-related biomarkers.

(E) Univariate Cox regression analysis comparing the prognostic value of CCL20 and the comparison biomarkers in the TCGA cohort.

Figure S6. Subgroup features and single-cell expression pattern of the CCL20/CCR6 axis.

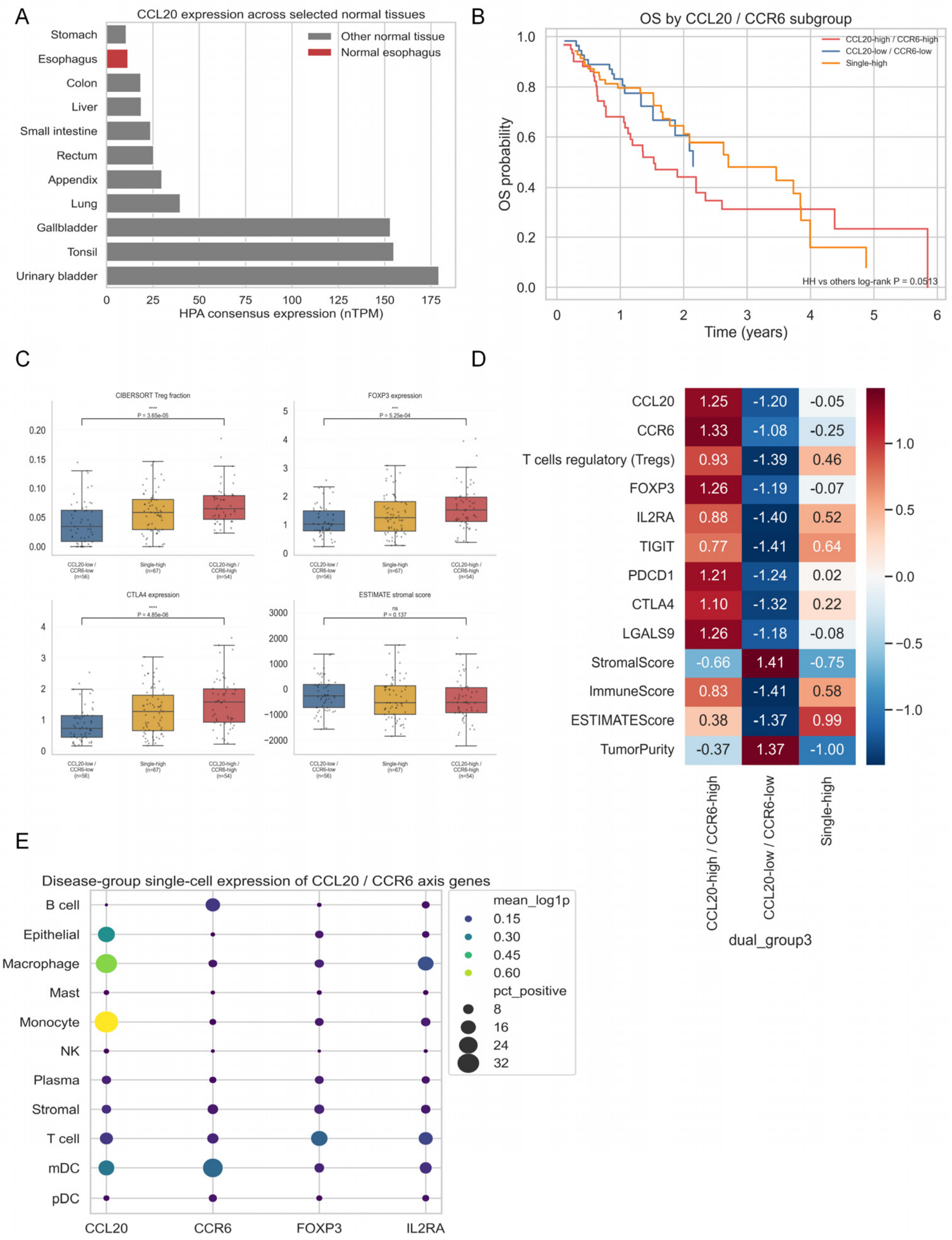

(A) CCL20 expression across selected normal tissues from the HPA database, with normal esophagus highlighted in red.

(B) Overall survival (OS) curves of patient subgroups stratified by CCL20 and CCR6 expression.

(C) Comparison of Treg fraction, FOXP3 expression, CTLA4 expression, and stromal score among subgroups.

(D) Heatmap of CCL20/CCR6 axis-related immune and microenvironmental features across subgroups.

(E) Dot plot showing CCL20, CCR6, FOXP3, and IL2RA expression across major cell types in the disease-group single-cell dataset. Dot size indicates the percentage of positive cells, and color indicates mean expression level.

Figure S7. Cross-cohort bulk co-expression validation of CCL20 virtual-knockout-associated genes.

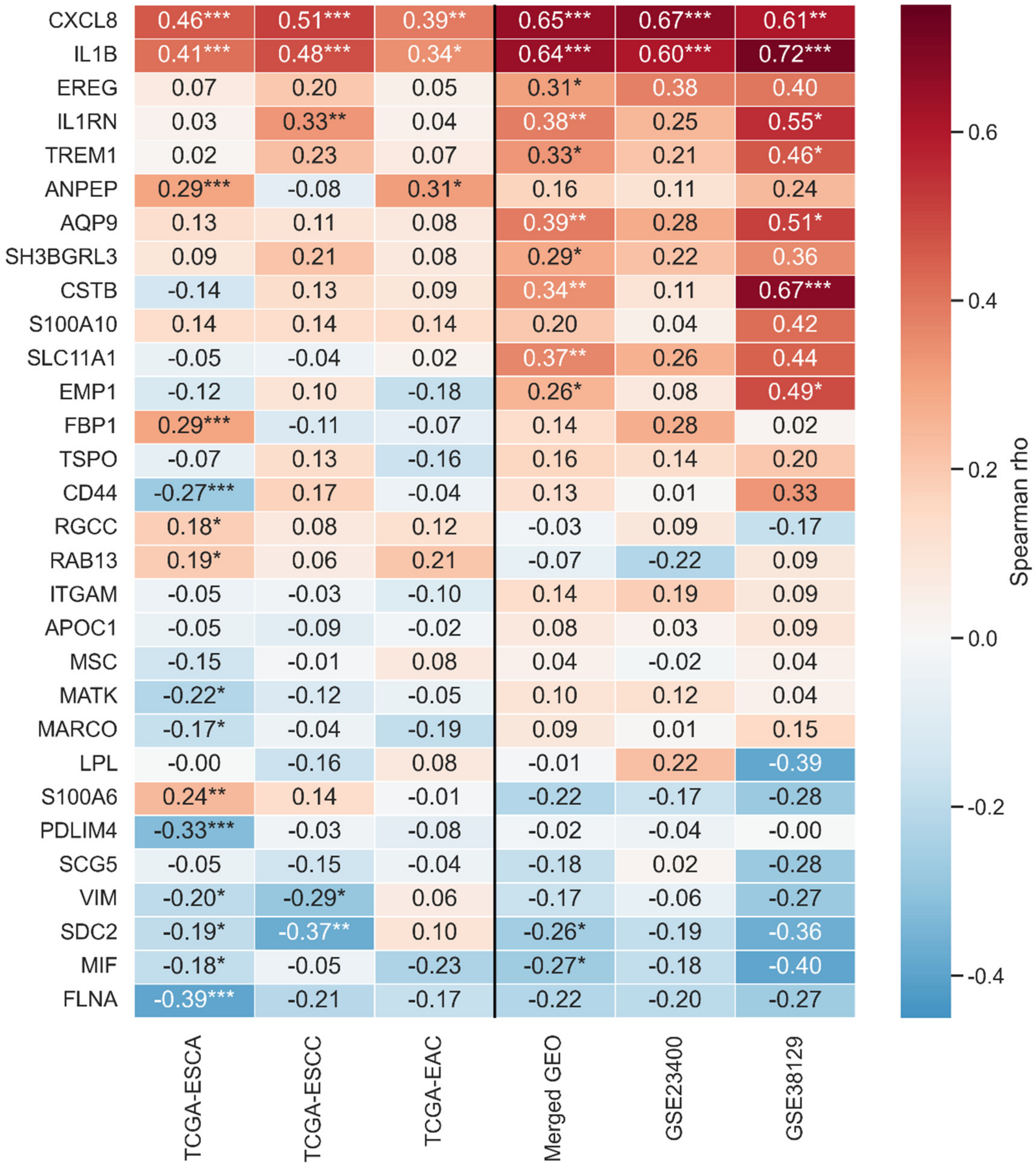

Heatmap showing Spearman correlation coefficients between CCL20 and scTenifoldKnk-identified virtual-knockout-associated genes across multiple ESCA bulk cohorts, including TCGA-ESCA, TCGA-ESCC, TCGA-EAC, merged GEO, GSE23400, and GSE38129. Colors indicate the direction and strength of correlation, numbers represent Spearman rho values, and asterisks denote statistical significance. Overall, CXCL8 and IL1B showed consistent positive correlations with CCL20 across independent cohorts, suggesting that they represent stable components of the CCL20-associated inflammatory network.

Supplementary Methods

Software environment and R package versions

All statistical analyses and visualizations were performed in R version 4.4.2. The main R packages used in this study included limma (version 3.62.2), WGCNA (version 1.73), ggplot2 (version 3.5.2), ggrepel (version 0.9.6), pheatmap (version 1.0.13), ComplexHeatmap (version 2.22.0), circlize (version 0.4.16), survival (version 3.7-0), survminer (version 0.5.0), timeROC (version 0.4), pROC (version 1.18.5), and sva (version 3.54.0).

Additional packages used for data processing, visualization, and figure assembly included patchwork (version 1.3.1), data.table (version 1.17.8), reshape2 (version 1.4.4), ggpubr (version 0.6.0), gridExtra (version 2.3), scales (version 1.4.0), viridis (version 0.6.5), corrplot (version 0.95), cowplot (version 1.1.3), scatterplot3d (version 0.3-44), and RColorBrewer (version 1.1-3).

Differential expression analysis thresholds

For the merged GEO discovery cohort, differential expression analysis was performed using the limma package. Genes with an absolute log2 fold change |log2FC| > 1 and a Benjamini–Hochberg-adjusted P value, also referred to as the false discovery rate (FDR), < 0.05 were defined as significantly differentially expressed genes.

For validation in the TCGA-ESCA cohort, candidate genes were compared between tumor and normal tissues using the Wilcoxon rank-sum test. The same statistical thresholds, |log2FC| > 1 and FDR < 0.05, were applied to define significantly differentially expressed genes. Throughout the revised analyses, raw P values and multiple-testing-adjusted P values were clearly distinguished, and adjusted P values/FDR were reported where multiple comparisons were involved.

WGCNA parameter settings

Weighted gene co-expression network analysis was performed using the WGCNA package. Before network construction, the expression matrix was normalized using the normalizeBetweenArrays function. Genes with variance in the lowest 25% were removed to reduce noise from low-variability genes.

A signed co-expression network was constructed. Candidate soft-thresholding powers ranging from 1 to 30 were evaluated using the pickSoftThreshold function. A signed scale-free topology fit index of $R^2 \geqslant 0.85$ was predefined as the criterion for selecting the soft-thresholding power. In the merged GEO discovery cohort, power = 14 was the first value reaching this criterion, with a signed R^2 of 0.860; therefore, the final soft-thresholding power was set to 14.

Modules were detected using the dynamic tree-cutting method. The minimum module size was set to 50 genes, and highly similar modules were merged using a module eigengene dissimilarity threshold of merge cutHeight = 0.25.

GEO cohort inclusion and sample filtering

For the GEO discovery analysis, two paired esophageal cancer transcriptomic datasets were included: GSE23400 and GSE38129. GSE23400 contained 106 samples, including 53 tumor tissues and 53 matched adjacent normal tissues. GSE38129 contained 60 samples, including 30 tumor tissues and 30 matched adjacent normal tissues.

After merging the two datasets and performing batch-effect correction, the final GEO discovery cohort included 166 samples, comprising 83 tumor tissues and 83 matched normal tissues. Quality control was

performed using the goodSamplesGenes function. All GEO samples passed quality control, and no additional samples were excluded from the main GEO discovery analysis.

TCGA-ESCA sample inclusion and exclusion

For TCGA-ESCA expression validation, the original dataset contained 198 samples, including 184 primary tumor samples with sample type code 01, 13 normal tissue samples with sample type code 11, and 1 metastatic sample with sample type code 06.

For tumor–normal differential expression analysis, only primary tumor samples and normal tissue samples were included. Therefore, the single metastatic sample was excluded. To ensure consistency of gene annotation, entries beginning with ENSG that could not be reliably mapped to standard gene symbols were removed, and duplicated gene symbols were collapsed before downstream analysis.

Survival analysis cohort construction

For survival analysis, only TCGA primary tumor samples were retained. Expression data and clinical information were matched using the 12-character TCGA patient identifier. Duplicate clinical records were removed. Patients without matched expression data or missing key survival information, including follow-up time (futime) or survival status (fustat), were excluded.

After filtering and matching, a total of 177 patients with available expression profiles and complete survival outcome information were included in the prognostic analysis. Genes with extremely low expression variability, such as those with a standard deviation < 0.01, were excluded from subsequent gene-wise Cox regression and Kaplan–Meier survival analyses.
